# Evidence for Mito-Nuclear and Sex-Linked Reproductive Barriers between the Hybrid Italian Sparrow and Its Parent Species

**DOI:** 10.1371/journal.pgen.1004075

**Published:** 2014-01-09

**Authors:** Cassandra N. Trier, Jo S. Hermansen, Glenn-Peter Sætre, Richard I. Bailey

**Affiliations:** Centre for Ecological and Evolutionary Synthesis, Department of Biology, University of Oslo, Oslo, Norway; University of Cambridge, United Kingdom

## Abstract

Studies of reproductive isolation between homoploid hybrid species and their parent species have rarely been carried out. Here we investigate reproductive barriers between a recently recognized hybrid bird species, the Italian sparrow *Passer italiae* and its parent species, the house sparrow *P. domesticus* and Spanish sparrow *P. hispaniolensis*. Reproductive barriers can be difficult to study in hybrid species due to lack of geographical contact between taxa. However, the Italian sparrow lives parapatrically with the house sparrow and both sympatrically and parapatrically with the Spanish sparrow. Through whole-transcriptome sequencing of six individuals of each of the two parent species we identified a set of putatively parent species-diagnostic single nucleotide polymorphism (SNP) markers. After filtering for coverage, genotyping success (>97%) and multiple SNPs per gene, we retained 86 species-informative, genic, nuclear and mitochondrial SNP markers from 84 genes for analysis of 612 male individuals. We show that a disproportionately large number of sex-linked genes, as well as the mitochondria and nuclear genes with mitochondrial function, exhibit sharp clines at the boundaries between the hybrid and the parent species, suggesting a role for mito-nuclear and sex-linked incompatibilities in forming reproductive barriers. We suggest that genomic conflict via interactions between mitochondria and sex-linked genes with mitochondrial function (“mother's curse”) at one boundary and centromeric drive at the other may best explain our findings. Hybrid speciation in the Italian sparrow may therefore be influenced by mechanisms similar to those involved in non-hybrid speciation, but with the formation of two geographically separated species boundaries instead of one. Spanish sparrow alleles at some loci have spread north to form reproductive barriers with house sparrows, while house sparrow alleles at different loci, including some on the same chromosome, have spread in the opposite direction to form barriers against Spanish sparrows.

## Author Summary

Hybridization between two species has the potential to create a third, hybrid species. However this process, known as hybrid speciation, is thought to be unlikely because it requires reproductive barriers against both parent species to develop despite the barriers between parents being weak enough to allow for the formation of viable, fertile hybrids. The Italian sparrow, which occupies the entire Italian peninsula and some Mediterranean islands, is the product of past hybridization between house and Spanish sparrows and therefore represents one of the few documented cases of vertebrate hybrid speciation in nature. We show that reproductive barriers between Italian sparrows and their parent species exist and that genes on the sex (Z) chromosome and mitochondria are heavily involved. We suggest that speciation in this system may have been driven by dissociation of the sex (Z) chromosome into blocks of different parent species-specific genes, which have shifted alongside mitochondrial genes to form reproductive barriers where the hybrid now meets each of its parent species.

## Introduction

Hybridization between divergent populations has diverse impacts on evolution [1]–[3], including the rapid formation of hybrid species [1]–[6]. Homoploid hybrid speciation (HHS) is the process through which hybridization between two taxa results in a third, novel taxon that remains distinct by means of reproductive barriers against both parent taxa, without a change in number of chromosome sets. This mode of speciation is thought to be rare in nature as hybridization must be initiated by gene exchange between two taxa, but this gene exchange also subsequently reduces the likelihood of hybrid speciation occurring. Gene flow from the parents must be countered or reduced after initial contact despite complementary ploidy levels and weak initial isolation [2], [5].

In non-hybrid speciation involving species with chromosomal sex-determination, sex-linked genes have repeatedly been found to strongly influence reproductive isolation (RI) [7]–[10]. The prominent role of sex chromosomes as reproductive barriers between non-hybrid species is attributed to a fast rate of genetic divergence, exposure of recessive alleles to selection in the heterogametic sex, a predominance of genes with sexual functions, and high average linkage between the disproportionately many sex-linked genes involved in RI [7], [8]. However, an equally important role for sex chromosomes in HHS is far from settled. One of the most likely mechanisms for HHS is thought to be through transgressive segregation: the production of trait values outside the range of both parent taxa in the hybrids, allowing adaptation to ecological niches unavailable to those parent taxa [5], [11]. Whereas divergent selection on reproductive traits is expected to be heavily involved in non-hybrid speciation, increasing the influence of sex chromosomes, transgressive ecological adaptations are more likely to be autosomal and evolve under stabilizing selection in parents [8], [11]–[13]. Divergent selection produces extreme phenotypes when it leads to parent taxa being fixed for alleles with opposite effects at each locus. Hence in this situation, additive genetic variation leads to intermediate hybrid trait values [11], [13]. In contrast, divergence under stabilizing selection occurs through weakly selected turnover of alleles contributing to a trait with an intermediate optimum. This promotes divergence on autosomes [12], and leads to a mixture of loci fixed for alleles with both positive and negative effects on trait values in each taxon. Thus, through additive effects alone, hybrids with more positive- or negative-effect alleles than in either parent taxon will often be produced, leading to transgressive phenotypes [11].

**Figure 1:**
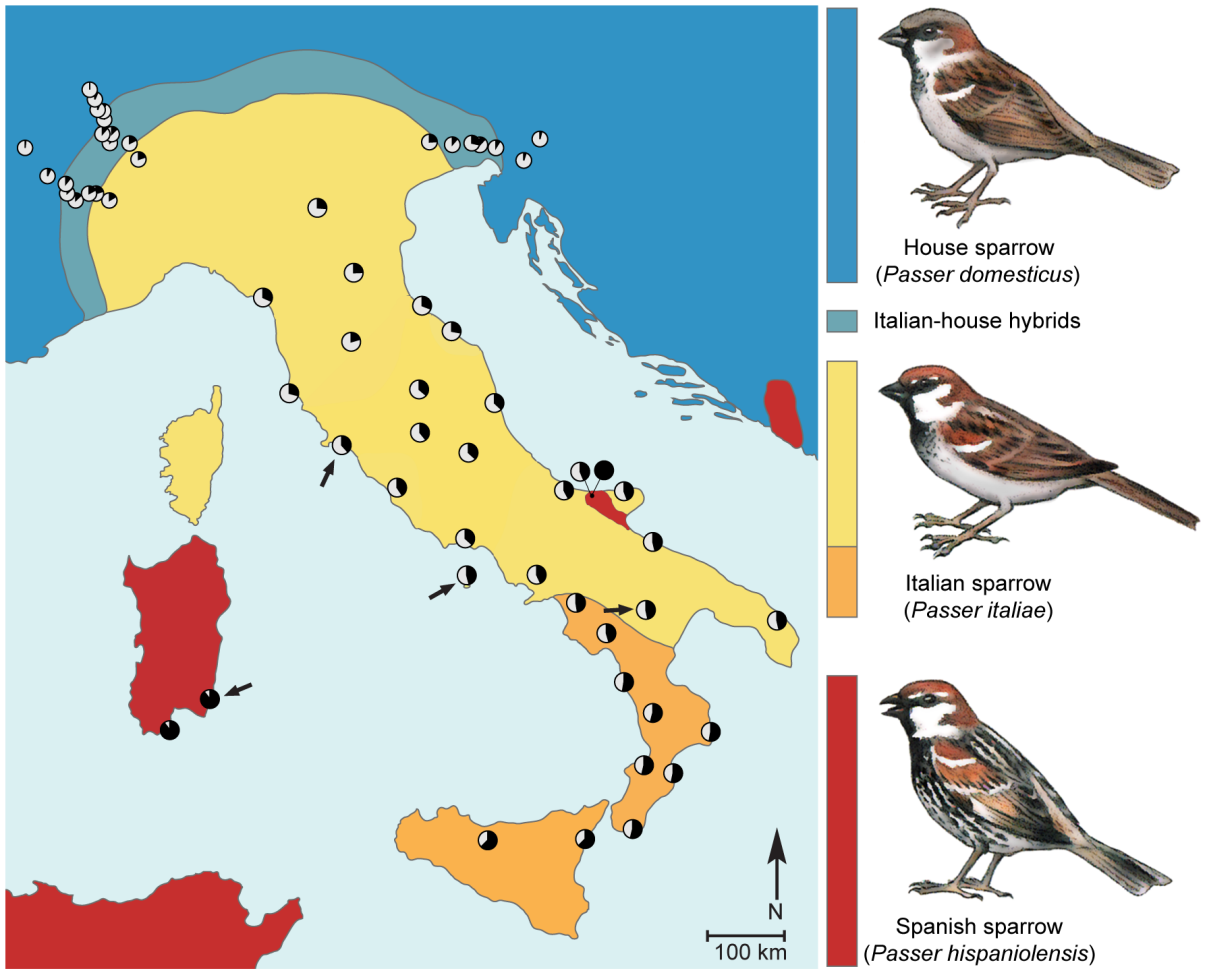
Phenotypic and genetic makeup of the hybrid Italian sparrow. Coloration of the map denotes phenotypic distribution as indicated by the bird drawings to the right of the map (blue: house sparrow, turquoise: Italian-house hybrids, yellow: typical Italian sparrow, orange: Italian sparrows with plumage intermediate between typical Italian and Spanish sparrows, red: Spanish sparrow). Bird drawings indicate species-specific male plumage characteristics of the three taxa [16]. Pie charts denote mean hybrid index at sampling localities where white and black color indicate house and Spanish sparrow genetic contribution, respectively. Locations with evidence of recent gene exchange between Spanish and Italian sparrows are indicated by arrows.

The study of RI in hybrid species systems can be complicated by a lack of geographical overlap between the hybrid and one or both of its parent species [5]. In *Passer* sparrows, however, the distribution of the hybrid Italian sparrow *Passer italiae* [14], [15] overlaps with those of both its parent species, the Spanish sparrow *P. hispaniolensis* and the house sparrow *P. domesticus* (Figure 1) [14] allowing for the study of reproductive barriers. The Italian sparrow is in contact with the house sparrow in a stable, narrow hybrid zone in the Alps [14], [16] and with the Spanish sparrow in a recently established sympatric zone in southeast Italy. In Sardinia, off the west coast of Italy, Spanish sparrows occur allopatrically (Figure 1). House and Spanish sparrows are themselves broadly, and often locally, sympatric across the entire Spanish sparrow range, remaining phenotypically distinct in all but a few locations [16]. Hence reproductive barriers exist and are typically effective in maintaining isolation between the parent species, but can be broken down to form viable hybrid populations and species.

Previous studies have indicated that Italian sparrows are almost fixed for house sparrow mitochondrial DNA [14], [15]. Moreover, two markers on the Z chromosome (birds are female-heterogametic with a ZZ/ZW sex chromosome system) were found to be fixed for the Spanish sparrow allele in one case (*CHD1Z*) and nearly fixed for the house sparrow allele in the other (*PLAA*), indicating strong mosaicism on the Z chromosome not paralleled by autosomal markers [15].

The evidence of Z chromosome mosaicism from existing studies [15] may indicate that Italian sparrow isolating mechanisms are more similar to those involved in non-hybrid speciation than would be expected given a strong influence of transgressive segregation. Furthermore, *CHD1Z* has been shown to be under divergent selection and associated with RI between several non-hybrid bird species pairs [15 and references therein]. For example, *CHD1Z* shows evidence of divergent selection in sympatric but not allopatric population comparisons of pied and collared flycatchers (*Ficedula hypoleuca* and *F. albicollis*) [17]. Should *CHD1Z* prove to be an informative marker in Italian sparrows, it may be particularly likely to represent a functional variant directly involved in RI (or at least a linked marker within the same gene) rather than a linked marker within a neutral gene.

Evidence is accumulating that mito-nuclear interactions cause postzygotic isolation and are influential in speciation [18], [19]. Mitochondrial DNA also commonly shows strongly shifted clines relative to nuclear markers, and this is often attributed to adaptive introgression [20]. Such introgression can occur in the face of detrimental effects on males, a phenomenon known as “mother's curse” [21], [22]: selection in males has no direct effect on mitochondrial fitness due to maternal inheritance, causing a selective sieve allowing the excessive build-up of male-detrimental mutations. Hence, if “mother's curse” is acting in the Italian sparrow we would expect male-compensatory alleles to track the spread of house sparrow mitochondria and show concordant clines.

Here, we analyze species-informative single nucleotide polymorphism (SNP) markers, located within functional transcribed genes, across the breeding range of the Italian sparrow, as well as the contact zones with its parent species. We use a cline analysis approach [23] to identify candidate hybrid-parent RI genes, and hence to elucidate the mechanisms involved in HHS. In particular, we look for evidence of coincidence between nuclear and mitochondrial clines and discuss the possibility that they represent the outcome of mother's curse in a hybrid species, and whether this mechanism may be influential in hybrid speciation. We test whether markers with clines falling on current hybrid-parent species boundaries are disproportionately (i) Z-linked, thus showing similarities with non-hybrid speciation, or (ii) autosomal, suggesting differences in mechanisms of hybrid speciation relative to non-hybrid speciation and a greater influence of stabilizing selection and transgressive segregation.

## Results

We identified putatively parent species-diagnostic SNP markers through transcriptome sequencing of six individuals of each of the two parent species. After filtering for coverage, sufficient flanking sequence, genotyping success (>97%) and multiple SNPs per gene, we retained 86 species-informative, genic, nuclear and mitochondrial SNP markers from 84 genes for analysis. Using this marker set, we found the Italian sparrow to exhibit high levels of genomic admixture over the entire study area (Figure 1 and Table S1). We also found evidence for on-going but restricted gene exchange between Italian sparrows and house sparrows in the contact zone in the Alps (Figure S1), though no evidence for gene exchange between Italian and Spanish sparrows in the sympatric zone in southeast Italy (see below). However, STRUCTURE [24] analysis revealed evidence of migration between Italian sparrow populations on mainland Italy and Spanish sparrow populations on Sardinia. Early generation migrants were present in both locations indicating ongoing gene flow through dispersal events (Figure 1).

As gene flow was observed between the Italian sparrow and both parent species, we implemented a cline analysis framework to look for genes exhibiting steep clines, and therefore decreased gene flow at the species boundaries. The SNPs are within functional coding genes and hence any such clines may indicate a direct influence of the gene on RI. They may also, however, be neutral but closely linked to loci under selection. These genes nevertheless represent the most likely candidates to be involved in RI at this inferential stage of analysis.

Cline analysis is a method used to measure the steepness, shape and location of changes in allele frequency or locus-specific ancestry, as well as in quantitative traits [23], [25]. It is typically used to examine geographic clines where, given the assumption that the cline is maintained by a balance between dispersal into a hybrid zone and selection against hybrids [25]–[27], various parameters including the strength of selection acting on traits or loci can be estimated. Cline analysis is therefore useful for identifying loci involved in RI in hybrid zones [23]. However, many contact zones do not conform to the assumption of a dispersal/selection balance and show a more complex pattern of contact and changes in locus-specific ancestry or allele frequency. This has led to the emergence of genomic cline analysis, in which geographic distance is replaced by a ‘hybrid index’, and cline width and location represent the amount and bias of introgression at a locus into the foreign genomic background [23], [28]–[30]. With the caveat that use of genomic clines does not fully remove the influences of genetic drift and geographic structure alongside selection on introgression, these analyses can be employed on any geographic pattern of contact and so are amenable for use in studies of hybrid speciation.

**Figure 2:**
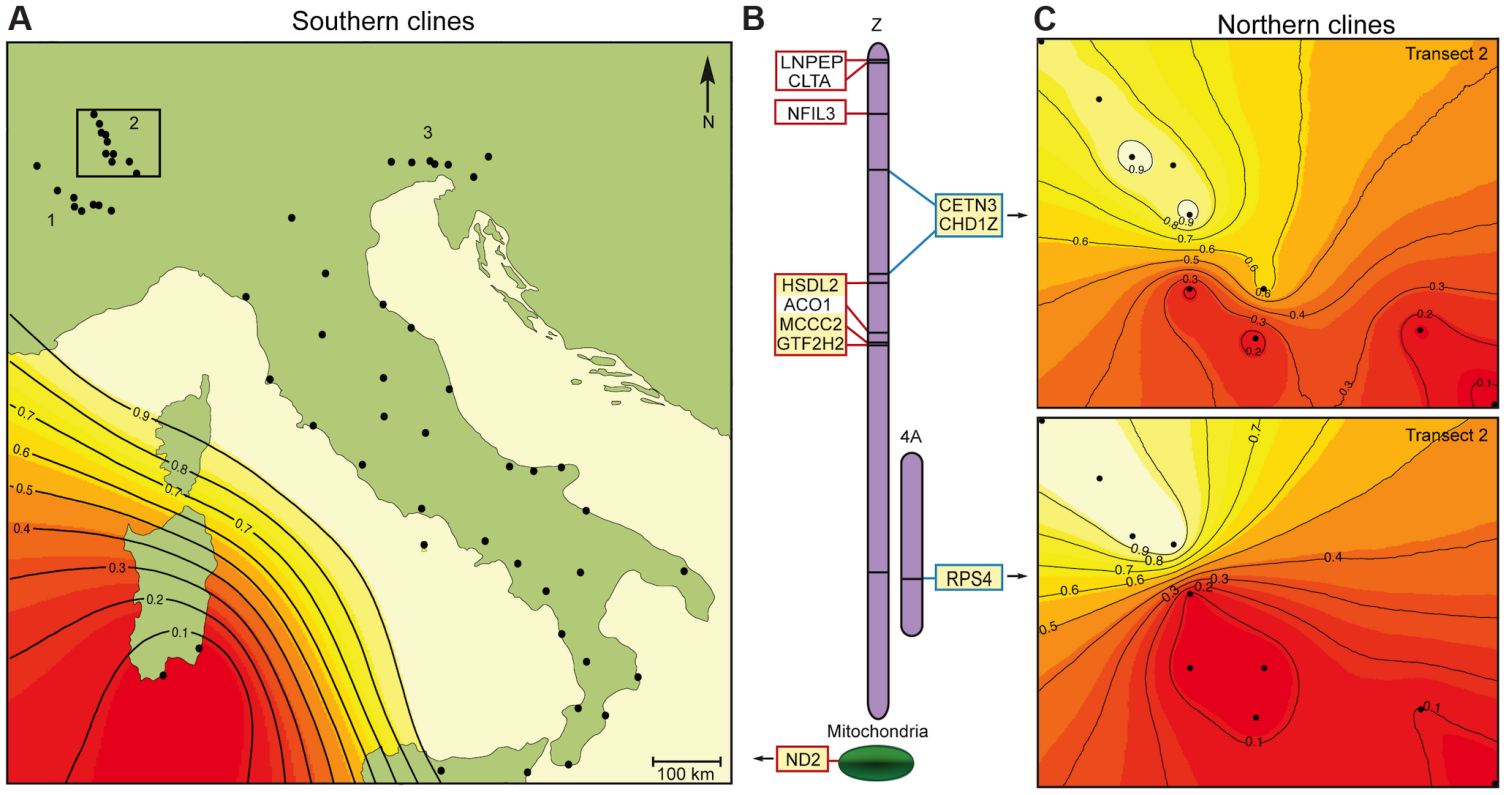
Genetic incompatibilities between the hybrid Italian sparrow and its parent species. (A) Representative geographic cline (*ND2*) for the mitochondrion and Z-linked genes shifting significantly between the Italian and Spanish sparrows of mainland Italy/Sicily and Sardinia. Colors refer to posterior likelihood of belonging to group corresponding to the Italian sparrow (>0.9, no color) relative to the Spanish sparrow (<0.1, red). The numbers refer to three transects through the Italian-house sparrow hybrid zone in the Alps. Black dots denote sampling locations. (B) Genomic location (in zebra finch) of genes inferred to be involved in hybrid-parent reproductive isolation. Blue outlines denote genes shifting significantly between the Italian and house sparrow, and red outlines denote genes shifting significantly between the Italian and Spanish sparrow. Markers highlighted in yellow have significantly steeper clines (significant β) than the neutral expectation according to a *BGC*-analysis (see main text) in addition to being significantly skewed towards either hybrid-parent species boundary (significant α), and hence represent the strongest candidate RI genes. Markers in white have significant α only. Chromosomal location for the Z-linked and Chr. 4A genes are indicated. (C) Geographic clines along transect 2 for the three genes shifting significantly in the Italian-house sparrow hybrid zone in the Alps. Upper panel shows results from the Z-linked genes *CHD1Z*/*CETN3*, lower panel shows results for the autosomal gene *RPS4*. Colors refer to posterior likelihood of belonging to group corresponding to the house sparrow (>0.9, white) relative to the Italian sparrow (<0.1, red). Black dots denote sampling locations.

**Figure 3:**
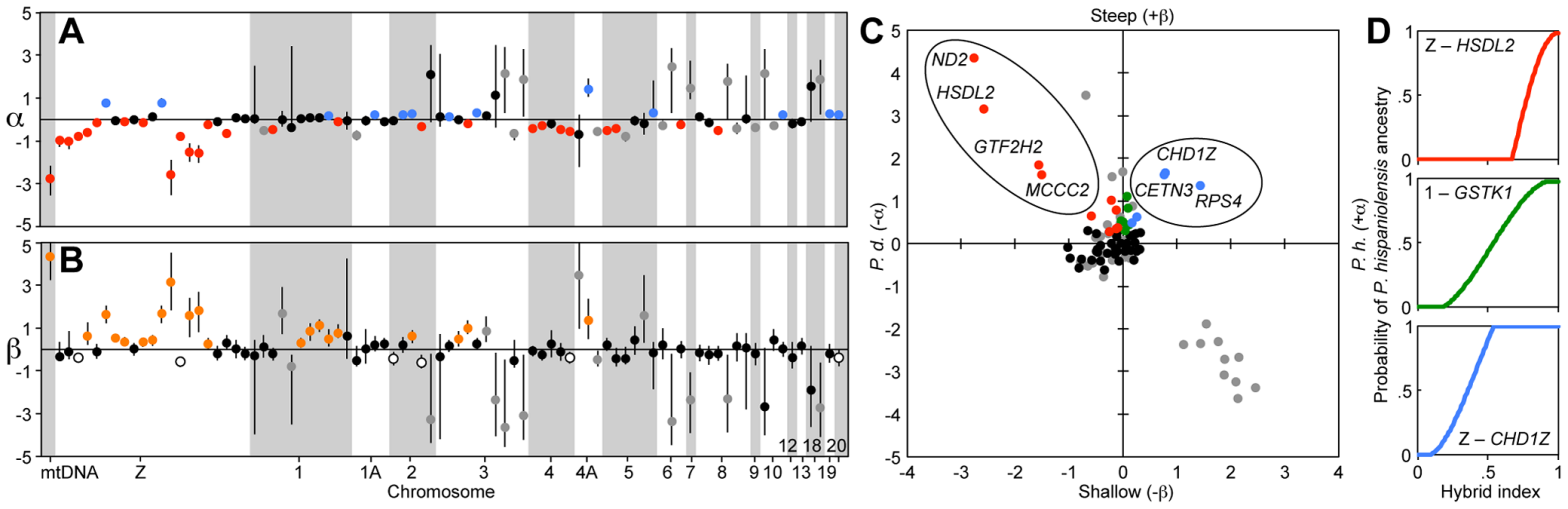
Candidate RI genes revealed by genomic cline analysis. (A–B) Markers are ordered along chromosomes as in Table S3. (A) *BGC* run 2 estimates of genomic cline center (α) with 95% credibility intervals for all 86 SNP markers. Red dots indicate markers with significant excess house sparrow ancestry, blue dots indicate markers with significant excess Spanish sparrow ancestry, grey dots indicate markers with either significant house or Spanish excess ancestry but where the difference in allele frequency between the parent species are below 0.5, and black dots indicate markers that do not differ from neutral expectations. (B) *BGC* run 2 estimates of genomic cline rate (β) with 95% credibility intervals for all 86 SNP markers. Orange dots indicate markers with clines significantly steeper than neutral expectations, white dots indicate markers with significantly shallower clines than neutral expectations, grey dots indicate markers with either significantly steeper or shallower clines than neutral expectations but where the difference in allele frequency between the parent species is below 0.5, and black dots indicate markers that do not differ from neutral expectations. (C) Genomic cline center (α) plotted against genomic cline rate (β). Red dots indicate markers that exhibit significant excess house sparrow (*P. d.*) ancestry, clines steeper than neutral expectations and where the difference in allele frequency between the parent species is greater than 0.5. The red dots that are encircled and named shift at the Italian-Spanish boundary and are hence candidate RI genes. Blue dots indicate markers that exhibit significant excess Spanish sparrow (*P. h.*) ancestry, clines steeper than neutral expectations and where the difference in allele frequency between the parent species is greater than 0.5. The blue dots that are encircled and named shift at the Italian-house boundary and are hence candidate RI genes. Green dots indicate markers that have allele frequency differences between the parent species greater than 0.5 and clines steeper than neutral expectations but do not exhibit excess house or Spanish sparrow ancestry. These markers are candidates for being incompatibilities within the Italian sparrow. Grey dots indicate markers in which the allele frequency difference between the parent species is less than 0.5. (D). Examples of *BGC* genomic clines representative of the marker categories described in panel (C). For illustrations of all clines, see Figure S2.

Bayesian genomic cline analysis (*BGC*) [29], [31] fits the Barton cline and estimates the parameters α (excess of house or Spanish alleles) and β (rate or steepness of cline), analogous to geographic cline center and width respectively [28], [30]. As there was some variation between runs, our *BGC* analysis revealed 31–35 genes to have excess house sparrow ancestry while 18–22 genes exhibited excess Spanish sparrow ancestry (Figure 2, Figure 3, and Figure S2). Furthermore, 25–27 genes exhibited steeper clines than neutral expectations (Figure 3, Figure S2) while 14–16 genes exhibited clines shallower than neutral expectations. Of the 25–27 genes with steeper clines than neutral expectations, 10 exhibited excess house sparrow ancestry, and another 5–8 exhibited excess Spanish sparrow ancestry. The remaining 8–10 genes exhibiting steep clines were not significantly shifted in either parental direction (Figure S2).

Combined *BGC* and geographical analysis using Geneland [32], [33] further revealed seven genes to exhibit abrupt allele frequency shifts and thus steep clines at the hybrid-parent range boundaries (Figure 2 and Figure 3, Table S2). Of these seven genes, five (i.e. 71.4%) were Z-linked (Figure 2 and Figure 3). Three of these Z-linked genes shifted at the Italian-Spanish boundary (Figure 2 and Figure S2) alongside mitochondrial *ND2*, whereas clines in two Z-linked and one autosomal gene were located at the Italian-house boundary (Figure 2, Figure 3 and Figure S3). These results indicate a mosaic pattern of introgression along the Z chromosome, as predicted by previous results [15]. There was a significant overrepresentation of Z-linkage among the genes exhibiting steep clines at the species boundaries considering that the Z chromosome holds about 3–7% of the genome of birds [9], [34], [35] (One-tailed binomial test: null probability based on flycatcher genome = 0.066, successes = 5, trials = 7, *P* = 2.35×10^−5^).

As observed in previous studies [14], [15], we found Italian sparrows to be nearly fixed for house sparrow mitochondrial haplotypes (Figure 2 and Figure 3; Table S2). Moreover, two of the three Z-linked genes that exhibit steep clines at the Italian-Spanish boundary are classified as nuclear-encoded mitochondrial proteins (*HSDL2* and *MCCC2*) [36], [37]. This is a significant overrepresentation of mitochondrial function compared to 8.3% in chickens [34], [37] (One-tailed binomial test: null probability = 0.083, successes = 2, trials = 3, *P* = 0.02; null probability data from The Gene Ontology Project's Gene Association file for *Gallus gallus*, GOC validation date: 4 December 2012, gaf-version 2.0 and The Gene Ontology Project's Gene Ontology file, date: 4 December 2012, cvs revision version 4708) among the nuclear genes shifting at this boundary. The Z-linked gene *HSDL2* exhibited a near-identical pattern of fixation for house sparrow alleles in the Italian sparrow as the mitochondrial marker *ND2* (Figure 3; Table S2).

In three transects through the hybrid zone in the Alps, the Z-linked markers *CETN3* and *CHD1Z* and autosomal marker *RPS4* exhibited the steepest clines (Figure 2 and Figure 3). Unlike in other bird species [9], [34], [35], *CHD1Z* and *CETN3* appear to be tightly linked in sparrows (Figure S4). Outlier analyses indicated that *CHD1Z* but not *CETN3* is a candidate for being under divergent selection (Figure S5).

The three markers *MCCC2*, *GTF2H2* and *HSDL2* also show evidence of statistical association, although *HSDL2* is predicted to be a long physical distance from the other two based on the zebra finch genome (Figure S4). A conservative estimate can therefore be made that two out of four sets of markers (*ND2*, *RPS4*, one from *CHD1Z*/*CETN3* and one from *HSDL2*/*MCCC2*/*GTF2H2*) with steep clines on range boundaries are Z-linked. This remains a significant overrepresentation of Z-linked markers (One-tailed binomial test: null probability = 0.066, successes = 2, trials = 4, *P* = 0.02).

While the steepest genomic clines were found for markers with major geographic clines at the Italian sparrow range boundaries (Figure 2 and Figure 3; Figure S2, Table S2), clines steeper than neutral expectations were also found within the Italian sparrow's range in a number of both autosomal and Z-linked genes (Figure 3c and Figure S2), some of which were also significantly shifted (significant α; Figure 3 a–c, Figure S2). Seven other markers were strongly shifted towards an excess of Spanish sparrow alleles, but had clines much shallower than the rest (Figure 3 a–c). Not all of the α and β estimates for these markers were significant however, and the parental allele frequency difference was <0.5 in every case. With such a low parental allele frequency difference and significantly shallow rather than steep clines, we do not consider these as potential RI genes. However, this does not rule out other forms of selection on these loci within the hybrid species.

Whereas Sardinian Spanish sparrows show evidence of on-going introgression from Italian sparrows, Spanish sparrows in the recently established sympatric population in southeast Italy appear to be genetically pure (Table S1, ‘Lesina (Spanish)’). We found no difference in *F*_ST_ between these Spanish sparrows and sympatric Italian sparrows versus Spanish sparrows and nearby allopatric Italian sparrows (Table 1). Thus, there was no sign of gene flow in sympatry between Italian and Spanish sparrows.

**Table 1:** Test for gene exchange in sympatry.

	Lesina Spanish	Guglionesi	Lesina Italian
**Guglionesi**	**0.4163** ***		
**Lesina Italian**	**0.4105** ***	0.0025	
**Mass Montanari**	**0.3926** ***	0.0073	0.0041

FST values between Gargano peninsula populations of Spanish sparrows and sympatric (Lesina) and nearby allopatric Italian sparrows.

indicates highly significant genotypic divergence, *P*<0.0001.

## Discussion

### Reproductive barriers between the hybrid Italian sparrow and its parent taxa

In this study, we utilized the Italian sparrow's gene exchange and geographical overlap with both parent species, house and Spanish sparrows, to investigate hybrid-parent reproductive barriers using a cline analysis framework. Results of geographic and genomic cline analyses reveal that several markers with an excess of Spanish sparrow alleles within Italian sparrows are associated with RI at the house-Italian range boundary, and that several markers with house sparrow excess are associated with RI at the Spanish-Italian range boundary. A disproportionately high number of the markers showing steep clines at one or the other hybrid-parent species boundary are Z-linked, including when potential physical linkage is accounted for. Of these SNPs, which are all within functioning transcribed genes, the strongest candidates to be within genes directly involved in RI are those that exhibit the most extreme cline parameters. This includes *CHD1Z* (Z chromosome) and *RPS4* (chromosome 4A) at the Italian-house sparrow boundary in the Alps, and *HSDL2* (Z chromosome) and *ND2* (mitochondria) at the Italian-Spanish sparrow boundary between the Italian mainland and Sardinia. Furthermore, two of the three nuclear markers with steep clines coincident with that of the mitochondria at the Italian-Spanish boundary (*HSDL2* and *MCCC2*) are classified as nuclear-encoded mitochondrial proteins [36], [37]; a statistical overrepresentation. As outlier loci revealed by cline analysis may result from genetic drift rather than from selection on introgression [29], we note that a significant overrepresentation of sex-linkage and mitochondrial function among candidate RI genes as reported here is not expected to arise through drift.

Excessive sex linkage of RI supports the hypothesis that HHS in Italian sparrows is facilitated by divergent selection between the parent species. HHS differs from non-hybrid speciation in that an isolating mechanism is required against each parent. This may be aided by transgressive segregation leading to extreme hybrid phenotypes [5], [11]; a process promoted more by stabilizing selection in the parent species than by divergent selection [11], [13]. Stabilizing selection is more likely to produce transgressive hybrid phenotypes through purely additive effects due to the greater likelihood of complementary gene action [11]. While traits under divergent selection may also produce transgressive phenotypes in some circumstances, for example with epistasis, the emphasis on transgression in HHS represents a contrast to theories of non-hybrid adaptive speciation, in which genes under divergent selection are thought more likely to contribute to isolation [38], [39].The mechanisms promoting Italian sparrow HHS may therefore more closely resemble those involved in typical non-hybrid speciation. While transgression cannot be ruled out, Italian sparrows appear phenotypically intermediate between the parents and share the house sparrow's human-commensal niche.

We recognize that the genes with steep clines may represent neutral markers in linkage disequilibrium with the RI genes under selection (this is likely to be the case for the mitochondrial *ND2*, which is in linkage disequilibrium with all other mitochondrial genes), rather than being directly involved in RI. In the case of *RPS4*, there is no *a priori* expectation that it should be involved in RI. However this gene, the sole autosomal representative associated with hybrid-parent isolation, is found on chromosome 4A in zebra finch [35]. This chromosome is orthologous to the mammalian X chromosome and *RPS4* is in fact X-linked in Eutherian mammals [40]. Chromosome 4A appears to be enriched for genes with properties similar to sex-linked genes, including the avian homolog to the human sex-determining *SRY*, and about one third of this chromosome has even translocated to the sex chromosomes in the whole avian superorder Sylvioidea [40]. Hence, the sole autosomal gene with a significantly steep cline on a species boundary may be linked to genes with properties more typical of sex-linked genes.

We hypothesized that *CHD1Z* may directly influence RI because it has been previously highlighted as a candidate speciation gene in other bird systems [15 and references therein]. This therefore represents a stronger RI gene candidate than *RPS4* or *CETN3* on the Italian-house boundary. Though *CHD1Z* and *CETN3* appear be closely linked to the same RI locus, our outlier analysis indicates *CHD1Z* is under divergent selection while *CETN3* is not, and is therefore a more likely candidate to be the RI locus. *CHD1* (the generic name for this gene in all organisms, represented by divergent Z-linked and W-linked copies in birds) is a chromatin-remodeling factor potentially affecting the expression of many genes [41]. Of particular interest is its essential role in chromosome centromere localization [42]. “Centromeric drive” is a proposed mechanism of intra-genomic conflict potentially causing rapid evolution of incompatibilities and speciation [43]. In this process, male-detrimental centromere drivers causing biased meiosis lead to selection for compensatory mutations in centromeric proteins, potentially including *CHD1*. Centromeric drive has been proposed as an important mechanism in the non-hybrid speciation of pied and collared flycatchers [9], in which *CHD1Z* also shows evidence of involvement in RI [17]. This adds weight to the argument that *CHD1Z* represents a candidate RI gene, maintaining parapatric differentiation between hybrid Italian sparrows and the house sparrow.

We also hypothesized a role for mito-nuclear interactions, and in particular the tracking of spreading mitochondrial variants by nuclear male ‘restorer’ genes, compensating for ‘mother's curse’. The overrepresentation of nuclear genes with a mitochondrial function on the Italian-Spanish sparrow boundary provides some support for this hypothesis. However, only one of these genes, *HSDL2*, shows a cline almost as steep as mitochondrial *ND2*. While this may be a linked neutral marker, the fact that *HSDL2*'s protein product is located within mitochondria [37], [44] supports its candidacy as an RI gene. *HSDL2* is thought to be involved in fatty acid metabolism, although its exact functions are unknown [44].

Our results thus appear consistent with the influence of mito-sex chromosome conflict acting as a reproductive barrier at the Spanish-Italian sparrow boundary. Furthermore, due to the lack of global dosage compensation in birds, Z-linked genes typically have higher expression and – combined with the fact that Z chromosomes spend two thirds of their time in males - stronger fitness effects in males than females. Consequently, genes with male-specific fitness effects are overrepresented on the Z chromosome [8], [45]. We thus postulate that nuclear male-compensatory ‘restorer’ genes are most likely to occur on the Z chromosome, leading to reduced fitness in hybrids with mito-sex chromosome mismatches. *HSDL2* in fact has been shown to have higher expression levels in male than female chickens [46]. As an alternative to “mother's curse”, isolation through co-adaptation between nuclear and mitochondrial genes is also possible. Because natural selection can only act on such bi-directional co-evolution through female fitness effects, we propose that mito-nuclear co-adaptation should involve disproportionately many autosomal genes, as they spend equal time in both sexes and show no overall sex bias in gene expression [45]. Hence our results are more consistent with mito-nuclear conflict.

There is no evidence for hybridization between Spanish and Italian sparrows in the sympatric zone of southeast Italy, supporting previous results [14]. This suggests a role for prezygotic barriers between the two taxa. Spanish sparrows are much less associated with humans than house and Italian sparrows and occupy a different habitat, providing some habitat-dependent assortative mating [16]. We suggest that evolution of Italian sparrows towards the house sparrow human-commensal niche may have contributed to rapid development of prezygotic isolation with Spanish sparrows alongside, or even reinforced by, the aforementioned mito-nuclear postzygotic barrier.

### Reproductive barriers within the hybrid Italian sparrow

In addition to the steep clines at the species range boundaries, some genes exhibited clines steeper than the neutral expectation within the Italian sparrow's range. One possible interpretation of this result is that moving clines of incompatibility genes may have become trapped by environmental transitions or population density troughs before reaching the current hybrid-parent boundaries. In this way, intraspecific incompatibilities within a hybrid species may increase future diversification relative to non-hybrid species, in particular through the effect of divergence hitchhiking in promoting the build-up of novel isolating mechanisms surrounding pre-existing incompatibilities [47], [48]. Isolation by adaptation may be occurring in Italian sparrows [49], so moving clines of incompatibility genes may also have become trapped by association with niche differentiation [50]. Nevertheless, neutral processes cannot be ruled out. The spatial spread of a partially reproductively isolated taxon into the range of the other taxon may lead to neutral allele frequency clines at historical invasion wave fronts [51], although these would become more diffuse over time since the spread.

The Italian sparrow genome represents a mosaic, particularly on the Z chromosome, in which some Spanish sparrow alleles have spread to form reproductive barriers against house sparrows, while house sparrow alleles at different loci on the same chromosome have spread in the opposite direction to form barriers against Spanish sparrows. We envisage that HHS in the Italian sparrow may match the ‘mosaic genome hybrid speciation’ model [52] with the addition of secondary spatial spread of the hybrid genotype. Such discordant spread would most likely occur through selective sweeps, if one parental allele had a fitness advantage over the other in the mosaic genomic background. However, if strong selective sweeps occurred, some mechanism would be needed to cause them to stop at the current hybrid-parent boundaries. We note that these boundaries lie on major barriers to dispersal in the case of the *Passer* sparrows, and that beneficial alleles often do not sweep across the whole range of a species for a variety of reasons including geographical barriers.

### Conclusions

Using a cline analysis framework we have identified sets of candidate RI genes and genomic regions between the hybrid Italian sparrow and its parent species. These results support our predictions that mito-nuclear interactions and loci on the Z chromosome strongly influence RI. In this regard, we suggest that HHS in the Italian sparrow resembles non-hybrid speciation, and we would therefore predict that the same loci would be involved in RI between the parent taxa; house and Spanish sparrows. An important next step is therefore to replicate this study in a region of parental sympatry and hybridization [53].

## Materials and Methods

### Sample collection

Only males, which are diploid for the Z chromosome, were analyzed to avoid issues related to haplodiploidy of the Z chromosome. Blood samples from the three taxa (*n* = 612) were taken from 64 locations between 2007 and 2011 (Figure 1 and Table S1): Spanish sparrows (*n* = 142) from Badajoz, Spain, Sardinia, Italy and a Spanish/Italian sympatric zone in southeast Italy; allopatric house sparrows (*n* = 85) from Hradec Králové, Czech Republic and Oslo, Norway; Italian sparrows, Italian-house hybrids and parapatric house sparrows from the Italian peninsula and the Alps (*n* = 385). The sparrows were caught using mist nets. About 25 µl of blood was extracted by venipuncture of a brachial vein and stored in 1 ml of Queens lysis buffer. Appropriate catching and sampling permits were obtained for all sampling locations from the relevant authorities. DNA was isolated using Qiagen DNeasy 96 Blood and Tissue Kits (Qiagen N.V., Venlo, Netherlands) according to the manufacturer's instructions. For transcriptome sequencing, three house (Oslo, Norway) and three Spanish (Badajoz, Spain) sparrows of each sex were sampled in October 2010. Liver, heart and brain tissue samples were taken and stored on RNAlater (100 mg tissue in 1400 µl buffer) according to the manufacturer's protocol.

### Transcriptomic cDNA library preparation and sequencing

Total RNA isolation from pooled liver, heart and brain samples followed by normalized cDNA library preparation was performed by Vertis Biotechnologie AG, Freising, Germany. Total RNA was isolated from the cell powders using the mirVana RNA kit (Ambion) including an on-column DNase treatment. From the total RNA samples, poly(A)+ RNA was prepared and fragmented with ultrasound (1 pulse of 30 sec at 4°C). First-strand cDNA was synthesized from the fragmented RNA using a N6 randomized primer and M-MLV RNaseH-reverse transcriptase. 454 adapters A and B were ligated to the 5′ and 3′ ends of the cDNA. The cDNA was amplified with PCR using a proof reading enzyme. Normalization was carried out by one cycle of denaturation and reassociation of the cDNA, resulting in N1-cDNA. Reassociated ds-cDNA was separated from the remaining ss-cDNA (normalized cDNA) by passing the mixture over a hydroxylapatite column. After hydroxylapatite chromatography, the ss-cDNA was PCR amplified. For GS FLX Titanium sequencing, the cDNA in the size range of 450–700 bp was eluted from preparative agarose gels. The resulting cDNA was double stranded, and had a size of about 450–700 bp. The six samples from each species were pooled in equal amounts and pyrosequenced on a Roche GS FLX Titanium sequencer at the Norwegian Sequencing Center using the manufacturer's protocol.

### Sequence alignment and SNP mining

The house sparrow reads were aligned and mapped against the zebra finch genome, and reads of both species were then mapped against the resulting contigs, providing a list of potential species-informative SNPs. Species-diagnostic SNPs from the twelve samples were chosen and subsequently filtered for those without sufficient flanking sequence for PCR-primer design. Genes were annotated by blasting against the zebra finch and chicken genomes. Two exceptions were SNPs within *CHD1Z* and *ND2* genes, which were genotyped using existing primers [15]. The two *CHD1Z* SNPs in this initial set are within an intron [15]. The genomes of *Passer* sparrows have so far not been mapped. Genomic locations of the various markers were therefore inferred based on the Zebra finch *Taeniopygia guttata* genome [35].

### Genotyping

Multiplex sets of PCR primers were designed and all individuals genotyped at each SNP locus using the Sequenom MassARRAY system at CIGENE, Norwegian University of Life Sciences, Ås, Norway. A total of 124 putatively diagnostic SNP markers from 107 different genes were genotyped successfully. Statistical analyses were carried out on a subset of 86 species informative SNPs after further filtering (Table S3). This involved removing SNPs with <97% genotyping success over all samples, plus removing all but one SNP from each gene (except in *APC* and *A2ML1*, in which two markers were included due to marked differences in parental allele frequencies).

### Detection of gene exchange

Recent migration between Spanish sparrows on Sardinia and Italian sparrows was identified using the USEPOPINFO model in STRUCTURE [22], with 100,000 iterations, a burnin of 50,000, GENSBACK set to three generations and the rest of the settings as default. A hybrid index value [54] was calculated for each individual, based on the 86 SNPs. In the Alps transects, the presence of many individuals with intermediate hybrid index indicated hybridization with house sparrows (Figure S1).

### Detection of SNPs associated with reproductive isolation

If genotype frequencies for individual SNPs change more sharply with changing hybrid index than a neutral expectation, this indicates a potential association with reproductive isolation. Examination of individual SNP cline width with respect to hybrid index was carried out using Bayesian genomic clines method as implemented in *BGC* [31]. Spanish sparrows from Badajoz and Gargano, and house sparrows from Oslo and Hradec Králové, were used to indicate parental genotype frequencies (Table S3). For the genomic clines analyses, all individuals from the Alps, the Italian peninsula (excluding Spanish sparrows from the southeast Italian sympatric zone), Sicily and Sardinia were pooled into one admixed population. Genotypes at the mitochondrial *ND2* locus were coded as diploid homozygote as *BGC* failed to run with a haploid marker included. Three independent runs with 100,000 iterations each were run with the first 25,000 iterations discarded as burnin, MCMC samples thinned by recording every fifth value, while the rest of the *BGC* settings were as default. SNPs were identified as significantly deviating from null expectations when the 95% credibility intervals of the cline parameters α and β did not cross zero. Once candidate SNPs were chosen using the genomic clines approach, geographic locations of sharp changes in allele frequency for each SNP were identified in GENELAND [32],[33] using the uncorrelated allele frequency model and allowing 1–10 clusters. Each SNP was run three times at 3 million iterations with a burn-in of 200. For some SNPs Geneland identified more than two geographic clusters, indicating multiple rapid changes in allele frequency. In these cases the main cline was determined to be on a species boundary if Geneland identified a cline on that boundary and *BGC* indicated a significantly shifted cline center (significant α) (Figure 2 and Figure S2). We further narrow our focus primarily to markers that also have significantly steep clines (significant positive β) in all three *BGC* runs (Figure 2 and Figure S2).

### Linkage disequilibrium

On top of using the zebra finch genome to identify gene location, genetic linkage was estimated using GENEPOP [55] to calculate a *P* value for genotypic disequilibrium between every SNP pair and by employing a Fisher test to combine probabilities across all populations in the Italian peninsula, the Alps and Sardinia (Figure S4).

### Population divergence in sympatric Italian and Spanish sparrows

To assess if there is introgression between sympatric Italian and Spanish sparrows, *F*_ST_ values and genotypic differentiation were calculated between four populations in southeast Italy (Lesina, which is a sympatric population of Italian and Spanish sparrows, and the nearby allopatric Italian sparrow populations of Mass. Montanari and Guglionesi) in GENEPOP [55] using males and the chosen 86 SNPs. *F*_ST_ estimates were calculated between i) the Italian sparrows from each population and ii) the Spanish sparrows in Lesina and the Italian sparrows in each of the three populations. A shift in *F*_ST_ towards or away from Spanish sparrows in Italian sparrows from Lesina, relative to the two allopatric populations, would indicate introgression or displacement respectively.

### Outlier tests for divergent selection

BAYESCAN [56] and LOSITAN [57] are softwares that implement methods to test for evidence of both divergent and balancing selection through *F*_ST_ outlier analysis of molecular markers. We used both softwares to determine which of *CHD1Z* and *CETN3*, two highly linked markers, showed stronger evidence for selection in the Alps house-Italian sparrow hybrid zone. Data from all 85 nuclear SNPs (haploid mtDNA markers cannot be run alongside diploid markers, and *ND2* is invariant in the Alps) were used and the three transects were pooled, excluding sites with just a single individual sampled. For LOSITAN the options ‘neutral mean *F*_ST_’ and ‘force mean *F*_ST_’ were chosen, along with the infinite alleles mutation model and 50 k simulations. For BAYESCAN, default settings were used.

### Ethics statement

Handling of birds were conducted according to guidelines approved by the relevant authorities in the respective countries (Museum National d'Histoire Naturelle, Centre de Recherches sur la Biologie de Populations d'Oiseaux, Paris (France), Institute for Environmental Protection and Research – ISPRA (Italy), Consejería de Industria, Energía y Medio Ambiente (Spain), Norwegian Food Safety Authority (Norway), Ministrstvo za okolje in proctor, Agencija Republike Slovenije za okolje (Slovenia) and Bundesamt für Umwelt BAFU, Abteilung Artenmanagement (Switzerland)).

## Supporting Information

Figure S1 Individuals with intermediate crown color have already been reported in the Alps contact zone [14], [16]. Further evidence of hybridization comes from a large proportion of individuals with hybrid index intermediate between house and Italian sparrows in the contact zone. Hybrid index for each individual is plotted against distance from the house sparrow end of each transect, along a) transect 1 (see Fig. 2), b) transect 2 and c) transect 3. d) Histogram of hybrid indices for populations at the house sparrow end of the three transects (‘House’), the rest of the transect collections (‘Hybrid zone’), and northern Italian populations away from the transects (‘Italian’).
(DOC)

Figure S2 Bayesian genomic clines for all 86 SNP markers. The legend in each panel indicates genomic location (chromosome number), SNP name and significance of cline center (α) and cline rate (β) parameters for three independent *BGC* runs. Significant excess house sparrow ancestry is indicated by H's, significant excess Spanish sparrow ancestry is indicated by S's, significantly steep clines are indicated by +'s and significantly narrow clines are indicated by −'s. The number of H's, S's, +'s and −'s indicate for how many runs the parameter estimates for a given marker differed from neutral expectations. When none of the runs differed from neutral expectations, this is indicated by ns. Red panels indicate markers that exhibit significant excess house sparrow ancestry and have clines steeper than neutral expectations, where the difference in allele frequency between the parent species is greater than 0.5 and for which Geneland revealed a shift at the Italian-Spanish sparrow boundary. These are hence candidate RI genes. Blue panels indicate markers that exhibit significant excess Spanish sparrow ancestry, clines steeper than neutral expectations, a difference in allele frequency between the parent species greater than 0.5 and with a shift at the Italian-house sparrow boundary. These are hence candidate RI genes. Green panels indicate markers that have allele frequency differences between the parent species greater than 0.5 and where the clines are steeper than neutral expectations but do not exhibit a major allele frequency cline on either hybrid-parent species boundary. These markers are candidates for being incompatibilities within the Italian sparrow. Only markers with significant ‘+’ on more than one *BGC* run are highlighted.
(DOC)

Figure S3 GENELAND geographic clines for Alps transects 1–3 (Fig. 2a), for the three loci exhibiting rapid changes in allele frequency coinciding with the hybrid zone between Italian and house sparrows (*CHD1Z*, *CETN3* and *RPS4*; see main text). Axes represent longitude (x axis) and latitude (y axis) in decimal degrees. Colors refer to posterior likelihood of belonging to the group corresponding to the house sparrow (>0.9, white) relative to the Italian sparrow (<0.1, red). Black dots denote sampling locations. *CHD1Z* and *RPS4* results for transect 2 are also represented in Fig. 2c.
(DOC)

Figure S4 Heat map of genotypic disequilibrium between Z-linked markers with significantly steep genomic clines. Marker positions in kbp are based on the zebra finch genome. Internal cell values indicate distances between genes in kbp. Cell colors represent genotypic disequilibrium, combining *P* values across all Italian peninsula and Sardinian populations. Dark blue *P* = 0; medium blue *P*<0.05; pale blue *P*<0.1.
(DOC)

Figure S5 *F*_ST_-outlier analyses. (A) LOSITAN graphic of test for evidence of divergent selection in the Alps, using the 85 nuclear SNPs and combining data from all three transects (not including populations represented by a single individual). SNPs in the red area are candidates for positive divergent selection. At a false discovery rate of 0.05 only *CHD1Z* and *RPS4* were significant outliers for divergent selection. (B) BAYESCAN graphic of test for evidence of divergent selection in the Alps, using the 85 nuclear SNPs and combining data from all three transects (not including populations represented by a single individual). The estimated alpha coefficient indicates the strength and direction of selection. A positive value of alpha suggests diversifying selection, whereas negative values suggest balancing or purifying selection. *CHD1Z* and *RPS4* (highlighted in red and named) were the only markers that were significant at a false discovery rate of 0.1.
(DOC)

Table S1 Sample population details.
(DOC)

Table S2 *F*_ST_ between Italian sparrow and its parent species where a steep cline exists on the hybrid-parent boundary, plus estimates of cline shift (α) and steepness (β). Values in shaded boxes are not significant (95% credibility intervals overlap with zero).
(DOC)

Table S3 Details of SNPs used in analyses.
(DOC)

## References

[1] The *Heliconius* Genome Consortium (2012) Butterfly genome reveals promiscuous exchange of mimicry adaptations among species. Nature 487: 94–98. 22722851 10.1038/nature11041 PMC3398145

[2] Abbot R, Albach D, Ansell S, Arntzen JW, Baird SJE, et al (2013) Hybridization and speciation. J Evol Biol 26: 229–246. 23323997 10.1111/j.1420-9101.2012.02599.x

[3] Sætre GP (2013) Hybridization is important in evolution, but is speciation? J Evol Biol 26: 256–258. 23324000 10.1111/jeb.12005

[4] Buerkle CA, Morris RJ, Asmussen MA, Rieseberg LH (2000) The likelihood of homoploid hybrid speciation. Heredity 84: 441–451. 10849068 10.1046/j.1365-2540.2000.00680.x

[5] Mallet J (2007) Hybrid speciation. Nature 446: 279–283. 17361174 10.1038/nature05706

[6] Buerkle CA, Rieseberg LH (2008) The rate of genome stabilization in homoploid hybrid species. Evolution 62: 266–275. 18039323 10.1111/j.1558-5646.2007.00267.x PMC2442919

[7] Sæther SA, Sætre G-P, Borge T, Wiley C, Svedin N, et al (2007) Sex chromosome–linked species recognition and evolution of reproductive isolation in flycatchers. Science 318: 95–97. 17916732 10.1126/science.1141506

[8] Qvarnström A, Bailey RI (2009) Speciation through evolution of sex-linked genes. Heredity 102: 4–15. 18781167 10.1038/hdy.2008.93

[9] Ellegren H, Smeds L, Burri R, Olason PI, Backström N, et al (2012) The genomic landscape of species divergence in *Ficedula* flycatchers. Nature 491: 756–760. 23103876 10.1038/nature11584

[10] Meyer M, Kircher M, Gansauge M-T, Li H, Racimo F, et al (2012) A high-coverage genome sequence from an archaic Denisovan individual. Science 338: 222–226. 22936568 10.1126/science.1224344 PMC3617501

[11] Rieseberg LH, Archer MA, Wayne RK (1999) Transgressive segregation, adaptation and speciation. Heredity 83: 363–372. 10583537 10.1038/sj.hdy.6886170

[12] Charlesworth B, Coyne JA, Barton NH (1987) The relative rates of evolution of sex chromosomes and autosomes. Am Nat 130: 113–146.

[13] Bailey RI, Eroukhmanoff F, Sætre G-P (2013) Hybridization and genome evolution II: Mechanisms of species divergence and their effects on evolution in hybrids. Curr Zool 59: 675–685.

[14] Hermansen JS, Sæther SA, Elgvin TO, Borge T, Hjelle E, et al (2011) Hybrid speciation in sparrows I: phenotypic intermediacy, genetic admixture and barriers to gene flow. Mol Ecol 20: 3812–3822. 21771138 10.1111/j.1365-294X.2011.05183.x

[15] Elgvin TO, Hermansen JS, Fijarczyk A, Bonnet T, Borge T, et al (2011) Hybrid speciation in sparrows II: a role for sex chromosomes? Mol Ecol 20: 3823–3837. 21762432 10.1111/j.1365-294X.2011.05182.x

[16] Summers-Smith JD (1988) The Sparrows: A study of the genus *Passer*. Calton: T & AD Poyser. 342 p.

[17] Backström N, Lindell J, Zhang Y, Palkopoulou E, Qvarnström A, et al (2010) A high-density scan of the Z chromosome in *Ficedula* flycatchers reveals candidate loci for diversifying selection. Evolution 64: 3461–3475. 20629730 10.1111/j.1558-5646.2010.01082.x

[18] Burton RS, Barreto FS (2012) A disproportionate role for mtDNA in Dobzhansky– Muller incompatibilities? Mol Ecol 21: 4942–4957. 22994153 10.1111/mec.12006

[19] Pritchard VL, Edmands S (2013) The genomic trajectory of hybrid swarms: outcomes of repeated crosses between populations of *Tigriopus californicus* . Evolution 67: 774–791. 23461327 10.1111/j.1558-5646.2012.01814.x

[20] Toews DPL, Brelsford A (2012) The biogeography of mitochondrial and nuclear discordance in animals. Mol Ecol 21: 3907–3930. 22738314 10.1111/j.1365-294X.2012.05664.x

[21] Frank SA, Hurst LD (1996) Mitochondria and male disease. Nature 383: 224. 8805695 10.1038/383224a0

[22] Gemmel NJ, Metcalf VJ, Allendorf FW (2004) Mother's curse: the effect of mtDNA on individual fitness and population viability. Trends Ecol Evol 19: 238–244. 16701262 10.1016/j.tree.2004.02.002

[23] Payseur BA (2010) Using differential introgression in hybrid zones to identify genomic regions involved in speciation. Mol Ecol Resour 10: 806–820. 21565092 10.1111/j.1755-0998.2010.02883.x

[24] Pritchard JK, Stephens M, Donnelly P (2000) Inference of population structure using multilocus genotype data. Genetics 155: 945–959. 10835412 10.1093/genetics/155.2.945 PMC1461096

[25] Barton NH, Hewitt GM (1985) Analysis of hybrid zones. Annu Rev Ecol Syst 16: 113–148.

[26] Barton NH, Hewitt GM (1989) Adaptation, speciation and hybrid zones. Nature 341: 497–503. 2677747 10.1038/341497a0

[27] Barton NH, Gale KS (1993) Genetic analysis of hybrid zones. In Harrison RG, editor. Hybrid zones and the evolutionary process. Oxford: Oxford Univ. Press. pp. 13–45.

[28] Szymura JM, Barton NH (1986) Genetic analysis of a hybrid zone between the fire-bellied toads, *Bombina bombina* and *B. variegate*, near Cracow in southern Poland. Evolution 40: 1141–1159. 10.1111/j.1558-5646.1986.tb05740.x 28563502

[29] Gompert Z, Buerkle CA (2011) Bayesian estimation of genomic clines. Mol Ecol 20: 2111–2127. 21453352 10.1111/j.1365-294X.2011.05074.x

[30] Fitzpatrick BM (2013) Alternative forms for genomic clines. Ecol Evol 3: 1951–1966. 23919142 10.1002/ece3.609 PMC3728937

[31] Gompert Z, Buerkle CA (2012) bgc: Software for Bayesian estimation of genomic clines. Mol Ecol Resour 12: 1168–1176. 22978657 10.1111/1755-0998.12009.x

[32] Guillot G, Estoup A, Mortier F, Cosson JF (2005) A spatial statistical model for landscape genetics. Genetics 170: 1261–1280. 15520263 10.1534/genetics.104.033803 PMC1451194

[33] Guillot G, Mortier F, Estoup A (2005) GENELAND: a computer package for landscape genetics. Mol Ecol Notes 5: 712–715.

[34] International Chicken Genome Sequencing Consortium (2004) Sequence and comparative analysis of the chicken genome provide unique perspectives on vertebrate evolution. Nature 432: 695–716. 15592404 10.1038/nature03154

[35] Warren WC, Clayton DF, Ellegren H, Arnold AP, Hillier LW, et al (2010) The genome of a songbird. Nature 464: 757–762. 20360741 10.1038/nature08819 PMC3187626

[36] Pagliarini DJ, Calvo SE, Chang B, Sheth SA, Vafai SB, et al (2008) A mitochondrial protein compendium elucidates complex I disease biology. Cell 134: 112–123. 18614015 10.1016/j.cell.2008.06.016 PMC2778844

[37] Ashburner M, Ball CA, Blake JA, Botstein D, Butler H, et al (2000) Gene Ontology: tool for the unification of biology. Nat Genet 25: 25–29. 10802651 10.1038/75556 PMC3037419

[38] Rundle HD, Nosil P (2005) Ecological speciation. Ecol Lett 8: 336–352.

[39] Schluter D (2009) Evidence for ecological speciation and its alternative. Science 323: 737–741. 19197053 10.1126/science.1160006

[40] Pala I, Hasselquist D, Bensch S, Hansson B (2012) Patterns of molecular evolution of an avian neo-sex chromosome. Mol Biol Evol 29: 3741–3754. 22826461 10.1093/molbev/mss177

[41] Agate RJ, Choe M, Arnold AP (2004) Sex differences in structure and expression of the sex chromosome genes *CHD1Z* and *CHD1W* in zebra finches. Mol Biol Evol 21: 384–396. 14660691 10.1093/molbev/msh027

[42] Okada M, Okawa K, Isobe T, Fukagawa T (2009) CENP-H–containing complex facilitates centromere deposition of CENP-A in cooperation with FACT and CHD1. Mol Biol Cell 20: 3986–3995. 19625449 10.1091/mbc.E09-01-0065 PMC2743618

[43] Henikoff S, Kami A, Malik SM (2001) The centromere paradox: stable inheritance with rapidly evolving DNA. Science 293: 1098–1102. 11498581 10.1126/science.1062939

[44] Kowalik D, Haller F, Adamski J, Moeller H (2009) In search for function of two human orphan SDR enzymes: Hydroxysteroid dehydrogenase like 2 (HSDL2) and short-chain dehydrogenase/reductase-orphan (SDR-O). J Steroid Biochem Mol Biol 117: 117–124. 19703561 10.1016/j.jsbmb.2009.08.001

[45] Ellegren H, Parsch J (2007) The evolution of sex-biased genes and sex-biased gene expression. Nat Rev Genet 8: 689–698. 17680007 10.1038/nrg2167

[46] Goerlich VC, Nätt D, Elfwing M, Macdonald B, Jensen P (2012) Transgenerational effects of early experience on behavioral, hormonal and gene expression responses to acute stress in the precocial chicken. Horm Behav 61: 711–718. 22465454 10.1016/j.yhbeh.2012.03.006

[47] Seehausen O (2004) Hybridization and adaptive radiation. Trends Ecol Evol 19: 198–207. 16701254 10.1016/j.tree.2004.01.003

[48] Via S, West J (2008) The genetic mosaic suggests a new role for hitchhiking in ecological speciation. Mol Ecol 17: 4334–4345. 18986504 10.1111/j.1365-294X.2008.03921.x

[49] Eroukhmanoff F, Hermansen JS, Bailey RI, Sæther SA, Sætre G-P (2013) Local adaptation within a hybrid species. Heredity 111: 286–292. 23695379 10.1038/hdy.2013.47 PMC3807261

[50] Barton NH, de Cara MAR (2009) The evolution of strong reproductive isolation. Evolution 63: 1171–1190. 19154394 10.1111/j.1558-5646.2009.00622.x

[51] Currat M, Ruedi M, Petit RJ, Excoffier L (2008) The hidden side of invasions: massive introgression by local genes. Evolution 62: 1908–1920. 18452573 10.1111/j.1558-5646.2008.00413.x

[52] Jiggins CD, Salazar C, Linares M, Mavarez J (2008) Hybrid trait speciation and *Heliconius* butterflies. Phil Trans R Soc B 363: 3047–3054. 18579480 10.1098/rstb.2008.0065 PMC2607310

[53] Rieseberg LH (1997) Hybrid origins of plant species. Annu Rev Ecol Syst 28: 359–389.

[54] Gompert Z, Buerkle CA (2010) INTROGRESS: a software package for mapping components of isolation in hybrids. Mol Ecol Resour 10: 378–384. 21565033 10.1111/j.1755-0998.2009.02733.x

[55] Rousset F (2008) GENEPOP'007: a complete re-implementation of the GENEPOP software for Windows and Linux. Mol Ecol Resour 8: 103–106. 21585727 10.1111/j.1471-8286.2007.01931.x

[56] Foll M, Gaggiotti O (2008) A genome-scan method to identify selected loci appropriate for both dominant and codominant markers: a Bayesian perspective. Genetics 180: 977–993. 18780740 10.1534/genetics.108.092221 PMC2567396

[57] Antao T, Lopes A, Lopes RJ, Beja-Pereira A, Luikart G (2008) LOSITAN: A workbench to detect molecular adaptation based on a *F_st_*-outlier method. BMC Bioinformatics 9: 323. 18662398 10.1186/1471-2105-9-323 PMC2515854

