# Supplementary figures and images for: Evidence for Mito-Nuclear and Sex-Linked Reproductive Barriers between the Hybrid Italian Sparrow and Its Parent Species
Source: PLoS Genet. 2014 Jan 9;10(1):e1004075. doi: 10.1371/journal.pgen.1004075 (PMC3886922; doi:10.1371/journal.pgen.1004075)

**
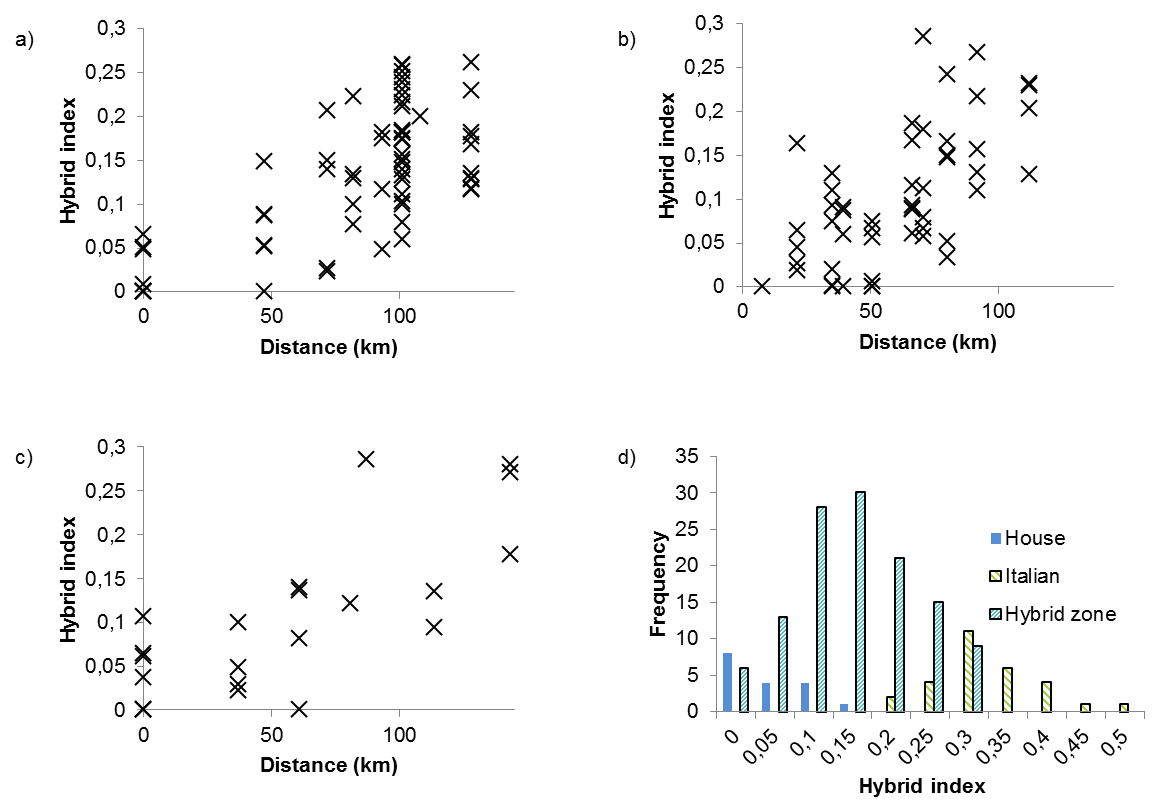
**

Supplement: Figure S1 — Individuals with intermediate crown color have already been reported in the Alps contact zone [14], [16]. Further evidence of hybridization comes from a large proportion of individuals with hybrid index intermediate between house and Italian sparrows in the contact zone. Hybrid index for each individual is plotted against distance from the house sparrow end of each transect, along a) transect 1 (see Fig. 2), b) transect 2 and c) transect 3. d) Histogram of hybrid indices for populations at the house sparrow end of the three transects (‘House’), the rest of the transect collections (‘Hybrid zone’), and northern Italian populations away from the transects (‘Italian’). (DOC) [file pgen.1004075.s001.doc]

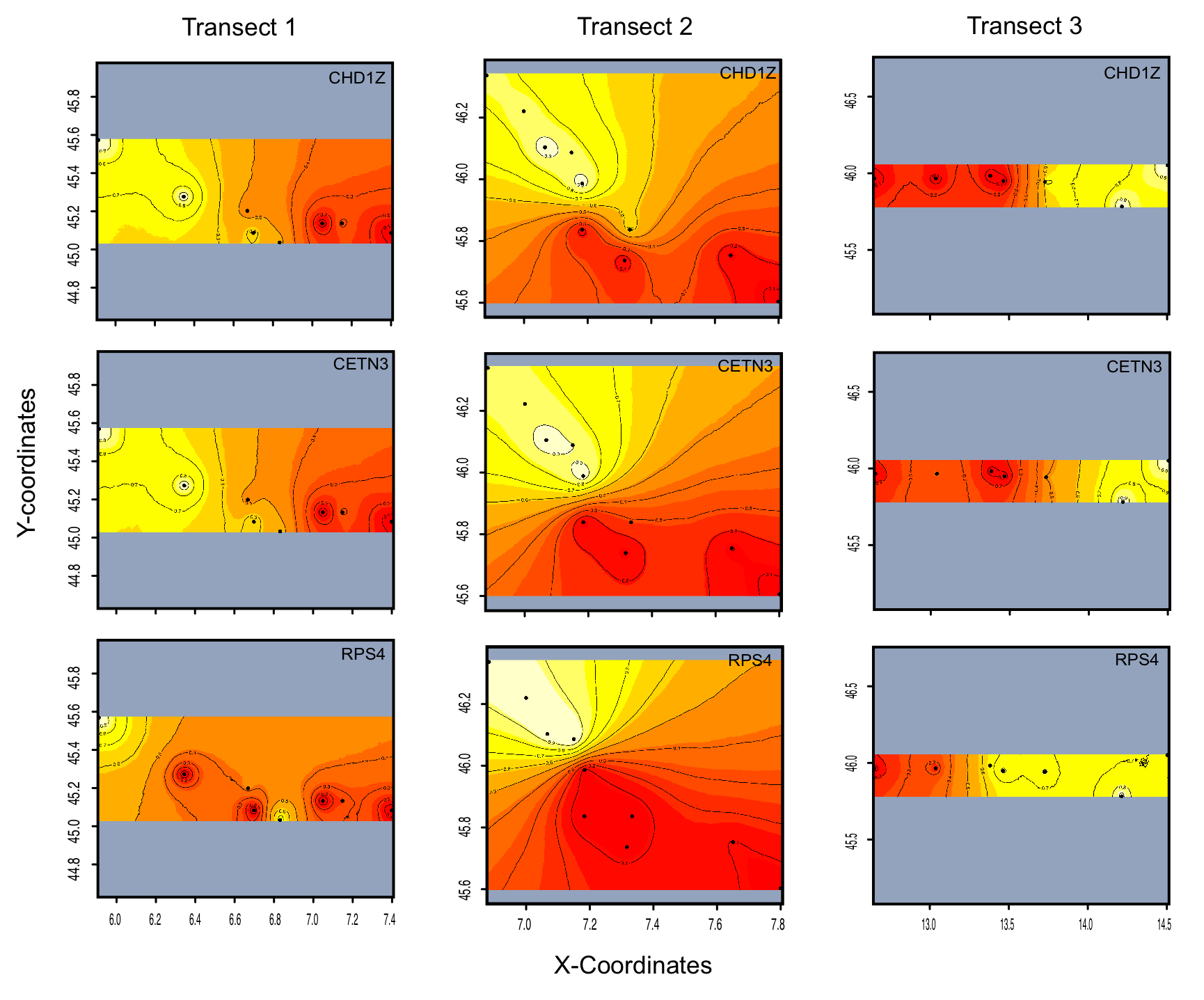

Supplement: Figure S3 — GENELAND geographic clines for Alps transects 1–3 (Fig. 2a), for the three loci exhibiting rapid changes in allele frequency coinciding with the hybrid zone between Italian and house sparrows (CHD1Z, CETN3 and RPS4; see main text). Axes represent longitude (x axis) and latitude (y axis) in decimal degrees. Colors refer to posterior likelihood of belonging to the group corresponding to the house sparrow (>0.9, white) relative to the Italian sparrow (<0.1, red). Black dots denote sampling locations. CHD1Z and RPS4 results for transect 2 are also represented in Fig. 2c. (DOC) [file pgen.1004075.s003.doc]
