# Supplementary material for: Evidence for Mito-Nuclear and Sex-Linked Reproductive Barriers between the Hybrid Italian Sparrow and Its Parent Species
Source: PLoS Genet. 2014 Jan 9;10(1):e1004075. doi: 10.1371/journal.pgen.1004075 (PMC3886922; doi:10.1371/journal.pgen.1004075)
Supplement: Table S1 — Sample population details. (DOC) [file pgen.1004075.s006.doc]

| **Table S1**. Sample population details. | | | | | | |
| --- | --- | --- | --- | --- | --- | --- |
| **Population** | **Decimal degrees, east** | **Decimal degrees, north** | **Sample size (n)** | | | **Mean hybrid index (min, max)** |
| Accettura | 16.16 | 40.49 | 27 | | | 0.47 (0.35, 0.61) |
| Aosta | 7.32 | 45.73 | 6 | | | 0.13 (0.03, 0.24) |
| Aquaviva-Picena | 13.81 | 42.95 | | 8 | 0.37 (0.31, 0.45) | |
| Assisi | 12.62 | 43.07 | 9 | | | 0.36 (0.28, 0.44) |
| Avigliana | 7.40 | 45.08 | 10 | | | 0.16 (0.12, 0.26) |
| Badolato Marina | 16.57 | 38.57 | 5 | | | 0.53 (0.49, 0.57) |
| Bardonecchia | 6.70 | 45.08 | 5 | | | 0.13 (0.08, 0.22) |
| Barletta | 16.28 | 41.32 | 6 | | | 0.46 (0.37, 0.55) |
| Bova Marina | 15.92 | 37.93 | 5 | | | 0.54 (0.48, 0.63) |
| Bussoleno | 7.15 | 45.13 | 1 | | | 0.2 (0.2, 0.2) |
| Castel di Guido | 12.28 | 41.90 | 6 | | | 0.41 (0.33, 0.51) |
| Chambery | 5.91 | 45.57 | 9 | | | 0.02 (0, 0.07) |
| Cormons | 13.47 | 45.95 | 1 | | | 0.12 (0.12, 0.12) |
| Cosenza | 16.25 | 39.30 | 3 | | | 0.54 (0.53, 0.57) |
| Crotone | 17.13 | 39.08 | 12 | | | 0.53 (0.38, 0.64) |
| Diamante | 15.83 | 39.68 | 9 | | | 0.53 (0.46, 0.6) |
| Eboli | 15.06 | 40.62 | 8 | | | 0.48 (0.37, 0.58) |
| Figline | 11.47 | 43.62 | 2 | | | 0.21 (0.15, 0.26) |
| Giardini-Naxos | 15.27 | 37.83 | 8 | | | 0.63 (0.51, 0.71) |
| Guglionesi | 14.92 | 41.91 | 13 | | | 0.42 (0.35, 0.49) |
| Lago di Burano | 11.38 | 42.40 | 6 | | | 0.38 (0.2, 0.78) |
| Lago di Fondi | 13.35 | 41.34 | 7 | | | 0.37 (0.25, 0.43) |
| L’aquila | 13.40 | 42.35 | 8 | | | 0.37 (0.27, 0.45) |
| Lecce | 18.17 | 40.35 | 5 | | | 0.46 (0.43, 0.51) |
| Lesina | 15.35 | 41.86 | 11 | | | 0.44 (0.37, 0.49) |
| Liddes | 7.18 | 45.98 | 6 | | | 0.03 (0, 0.07) |
| Ljubljana | 14.51 | 46.05 | 7 | | | 0.04 (0, 0.11) |
| Luni | 10.02 | 44.07 | 5 | | | 0.32 (0.21, 0.5) |
| Mantova | 10.80 | 45.16 | 1 | | | 0.26 (0.26, 0.26) |
| Manzano | 13.38 | 45.98 | 1 | | | 0.29 (0.29, 0.29) |
| Martigny | 7.07 | 46.10 | 7 | | | 0.06 (0, 0.13) |
| Mass. Montanari | 15.85 | 41.91 | 14 | | | 0.44 (0.32, 0.56) |
| Modane | 6.67 | 45.20 | 5 | | | 0.11 (0.02, 0.21) |
| Mondolfo | 13.10 | 43.75 | 3 | | | 0.28 (0.23, 0.34) |
| Montemaggiore Belsito | 13.76 | 37.85 | 6 | | | 0.63 (0.44, 0.75) |
| Oulx | 6.83 | 45.03 | 4 | | | 0.13 (0.05, 0.18) |
| Ozeljan | 13.73 | 45.94 | 4 | | | 0.09 (0, 0.14) |
| Ozzano D’Emilia | 11.47 | 44.44 | 1 | | | 0.25 (0.25, 0.25) |
| Pont Saint Martin | 7.80 | 45.60 | 4 | | | 0.2 (0.13, 0.23) |
| Pordenone | 12.65 | 45.97 | 3 | | | 0.24 (0.18, 0.28) |
| Postojna | 14.22 | 45.78 | 4 | | | 0.05 (0.02, 0.1) |
| Pula (Spanish) | 8.98 | 38.97 | 25 | | | 0.9 (0.82, 1) |
| Rimini | 12.57 | 44.06 | 14 | | | 0.31 (0.21, 0.44) |
| Saint-Rhémy-en-Bosses | 7.18 | 45.83 | 7 | | | 0.11 (0.06, 0.19) |
| Saint Vincent | 7.65 | 45.75 | 5 | | | 0.18 (0.11, 0.27) |
| San Priamo (Spanish) | 9.56 | 39.36 | 23 | | | 0.9 (0.78, 1) |
| Sanza | 15.55 | 40.25 | 10 | | | 0.46 (0.37, 0.55) |
| Scisciano | 14.49 | 40.92 | 8 | | | 0.43 (0.3, 0.5) |
| Sembrancher | 7.15 | 46.08 | 4 | | | 0.06 (0, 0.09) |
| Staz. Populonia | 10.54 | 43.00 | 6 | | | 0.29 (0.19, 0.39) |
| St Jean De Maurienne | 6.35 | 45.27 | 6 | | | 0.07 (0, 0.15) |
| St Maurice | 7.00 | 46.22 | 5 | | | 0.06 (0.02, 0.16) |
| Susa | 7.05 | 45.13 | 26 | | | 0.17 (0.06, 0.26) |
| Terni | 12.65 | 42.56 | 4 | | | 0.39 (0.34, 0.45) |
| Valpelline | 7.33 | 45.83 | 6 | | | 0.13 (0.06, 0.29) |
| Ventotene | 13.42 | 40.79 | 12 | | | 0.46 (0.26, 0.57) |
| Vibo | 16.10 | 38.68 | 4 | | | 0.54 (0.46, 0.6) |
| Vouvry | 6.88 | 46.33 | 1 | | | 0 (0, 0) |
| Zompicchia | 13.04 | 45.97 | 2 | | | 0.11 (0.09, 0.14) |
| Hradec Králové (house) | 15.88 | 50.57 | 27 | | | 0.007 (0, 0.05) |
| Oslo (house) | 10.74 | 59.91 | 58 | | | 0.001 (0, 0.06) |
| Badajoz (Spanish) | -7.22 | 38.65 | 86 | | | 0.99 (0.91, 1) |
| Lesina (Spanish) | 15.35 | 41.86 | 8 | | | 1 (1, 1) |
