# Supplementary material for: Evidence for Mito-Nuclear and Sex-Linked Reproductive Barriers between the Hybrid Italian Sparrow and Its Parent Species
Source: PLoS Genet. 2014 Jan 9;10(1):e1004075. doi: 10.1371/journal.pgen.1004075 (PMC3886922; doi:10.1371/journal.pgen.1004075)
Supplement: Table S2 — F ST between Italian sparrow and its parent species where a steep cline exists on the hybrid-parent boundary, plus estimates of cline shift (α) and steepness (β). Values in shaded boxes are not significant (95% credibility intervals overlap with zero). (DOC) [file pgen.1004075.s007.doc]

| **Table S2**. *F*_ST_ between Italian sparrow and its parent species where a steep cline exists on the hybrid-parent boundary, plus estimates of cline shift (α) and steepness (β). Values in shaded boxes are not significant (95% credibility intervals overlap with zero). | | | | | | | | | | |
| --- | --- | --- | --- | --- | --- | --- | --- | --- | --- | --- |
| **Locus** | **Boundary** | **FST^a^** | **α 1** | **α 2** | **α 3** | **Mean α** | **β 1** | **β 2** | **β 3** | **Mean β** |
| *ND2* | Spanish | 1.00 | -2.07 | -2.37 | -2.04 | -2.16 | 3.40 | 3.91 | 3.42 | 3.58 |
| *CETN3* | House | 0.21(W) 0.51(E) | 0.96 | 0.87 | 0.88 | 0.91 | 1.77 | 1.67 | 1.77 | 1.74 |
| *CHD1Z* | House | 0.19(W) 0.57(E) | 0.99 | 0.92 | 0.90 | 0.94 | 1.83 | 1.77 | 1.80 | 1.80 |
| *HSDL2* | Spanish | 0.97 | -2.01 | -2.31 | -2.38 | -2.23 | 2.44 | 2.90 | 2.90 | 2.75 |
| *MCCC2* | Spanish | 0.81 | -1.33 | -1.45 | -1.31 | -1.36 | 1.36 | 1.63 | 1.32 | 1.44 |
| *GTF2H2* | Spanish | 0.82 | -1.32 | -1.50 | -1.35 | -1.39 | 1.51 | 1.81 | 1.49 | 1.60 |
| *RPS4* | House | 0.31 | 1.57 | 1.53 | 1.41 | 1.50 | 1.31 | 1.42 | 1.22 | 1.32 |
| ^a^*F*_ST_ for *ND2* Estimated using GENEPOP. For *CHD1Z* AND *CETN3*, '(W)' is the two western transects; '(E)' is the eastern transect. Geneland identified 3 clusters in the northern Italian peninsula and Alps: One representing the 'house' half of the three transects, one representing the 'Italian' half of the two western transects, and a third representing the 'Italian' half of the eastern transect plus the Po Valley and north-central Italy. | | | | | | | | | | |
