# Supplementary material for: Evidence for Mito-Nuclear and Sex-Linked Reproductive Barriers between the Hybrid Italian Sparrow and Its Parent Species
Source: PLoS Genet. 2014 Jan 9;10(1):e1004075. doi: 10.1371/journal.pgen.1004075 (PMC3886922; doi:10.1371/journal.pgen.1004075)
Supplement: Table S3 — Details of SNPs used in analyses. (DOC) [file pgen.1004075.s008.doc]

| **Table S3**. Details of SNPs used in analyses. | | | | |
| --- | --- | --- | --- | --- |
| **Locus** | **Chromosome** | **Locus position Kbp (zebra finch)** | **Frequency of house sparrow allele in each parent species** | |
|  |  |  | **House sparrow** | **Spanish sparrow** |
| *ND2* | mtDNA | **-** | 1.000 | 0.022 |
| *LNPEP* | Z | 1650 | 1.000 | 0.000 |
| *CLTA* | Z | 2048 | 0.947 | 0.000 |
| *NFIL3* | Z | 6415 | 0.988 | 0.000 |
| *SECISBP2* | Z | 7215 | 0.918 | 0.005 |
| *ZCCHC6* | Z | 10697 | 0.882 | 0.011 |
| *CETN3* | Z | 12839 | 1.000 | 0.000 |
| *ZFAND5* | Z | 16367 | 1.000 | 0.000 |
| *APC_2* | Z | 21208 | 1.000 | 0.000 |
| *APC_6* | Z | 21208 | 0.835 | 0.000 |
| *REEP5* | Z | 21269 | 1.000 | 0.000 |
| *SNX2* | Z | 23313 | 0.900 | 0.000 |
| *CHD1Z* | Z | 24761 | 1.000 | 0.000 |
| *HSDL2* | Z | 25608 | 1.000 | 0.016 |
| *ACO1* | Z | 31875 | 1.000 | 0.027 |
| *MCCC2* | Z | 32227 | 1.000 | 0.027 |
| *GTF2H2* | Z | 32332 | 1.000 | 0.027 |
| *PIK3C3* | Z | 34838 | 0.965 | 0.086 |
| *TJP2* | Z | 57621 | 0.810 | 0.037 |
| *ADFP* | Z | 58329 | 1.000 | 0.011 |
| *MAP1B* | Z | 65941 | 0.888 | 0.269 |
| *TNPO1* | Z | 66268 | 0.876 | 0.011 |
| *CNKSR2* | 1 | 14015 | 0.512 | 0.144 |
| *MYCBP2* | 1 | 70107 | 0.482 | 0.016 |
| *NDFIP2* | 1 | 71316 | 0.818 | 0.117 |
| *CHORDC1* | 1 | 81730 | 0.900 | 0.683 |
| *TMEM135* | 1 | 82748 | 0.382 | 0.011 |
| *RSF1* | 1 | 87063 | 0.941 | 0.005 |
| *A2M* | 1 | 87885 | 0.988 | 0.021 |
| *A2ML1_2* | 1 | 87995 | 1.000 | 0.021 |
| *A2ML1_6* | 1 | 87995 | 0.882 | 0.021 |
| *GSTK1* | 1 | 89366 | 1.000 | 0.021 |
| *STAUR* | 1 | 93010 | 1.000 | 0.930 |
| *COG5* | 1A | 13709 | 0.135 | 0.005 |
| *SLC38A2* | 1A | 30337 | 0.747 | 0.366 |
| *VWF* | 1A | 62944 | 0.759 | 0.016 |
| *DYRK4* | 1A | 66520 | 0.935 | 0.074 |
| *LANCL2* | 2 | 32347 | 0.859 | 0.032 |
| *HypC2^a^* | 2 | 33014 | 0.655 | 0.032 |
| *CDC2l5* | 2 | 34253 | 0.965 | 0.069 |
| *PTPRM* | 2 | 104525 | 0.732 | 0.032 |
| *RB1CC1* | 2 | 115950 | 0.547 | 0.090 |
| *CRLS1* | 3 | 898 | 0.827 | 0.737 |
| *WDR92* | 3 | 3504 | 0.988 | 0.161 |
| *PPP3R1* | 3 | 3534 | 0.876 | 0.188 |
| *MIA3* | 3 | 8755 | 1.000 | 0.032 |
| *RRP15* | 3 | 10347 | 0.841 | 0.016 |
| *GNPAT* | 3 | 42061 | 0.429 | 0.005 |
| *Vps20* | 3 | 52254 | 0.500 | 0.027 |
| *SLC22A2* | 3 | 57706 | 0.196 | 0.016 |
| *PHF3* | 3 | 87982 | 0.824 | 0.415 |
| *YWHAQ* | 3 | 98885 | 0.429 | 0.032 |
| *G3BP2* | 4 | 1331 | 0.959 | 0.053 |
| *ANKRD17* | 4 | 1430 | 0.935 | 0.032 |
| *FAM13A1* | 4 | 1638 | 0.382 | 0.037 |
| *EFHA2* | 4 | 41418 | 0.647 | 0.027 |
| *STIM2* | 4 | 52243 | 0.912 | 0.038 |
| *SMARCA1* | 4A | 11621 | 0.859 | 0.830 |
| *RPS4* | 4A | 15541 | 0.853 | 0.016 |
| *TFIID1* | 4A | 19859 | 0.465 | 0.086 |
| *RASGRP1* | 5 | 30162 | 0.712 | 0.000 |
| *HECTD1* | 5 | 34222 | 0.673 | 0.011 |
| *ARHGAP5* | 5 | 34575 | 0.329 | 0 |
| *SMEK1* | 5 | 45219 | 0.875 | 0.586 |
| *BTBD7* | 5 | 46047 | 0.682 | 0.617 |
| *USP47* | 5 | ? | 0.918 | 0.382 |
| *CCAR1* | 6 | 1636 | 0.363 | 0.011 |
| *HypPC6^b^* | 6 | 16819 | 0.133 | 0.065 |
| *GSTO2* | 6 | 23909 | 0.694 | 0.069 |
| *OSBPL6* | 7 | 17903 | 0.406 | 0.165 |
| *RABGAP1l* | 8 | 188 | 0.582 | 0.016 |
| *UnTrC8* | 8 | 2210 | 0.547 | 0.027 |
| *LPPR1* | 8 | 8475 | 0.841 | 0.106 |
| *EPS15* | 8 | 21593 | 0.369 | 0.059 |
| *C8B* | 8 | 23643 | 0.153 | 0.021 |
| *PRRC2C* | 8 | ? | 0.406 | 0.011 |
| *ILKAP* | 9 | 4943 | 0.795 | 0.332 |
| *KIAA1370* | 10 | 8472 | 0.445 | 0.413 |
| *COPS2* | 10 | 10211 | 0.606 | 0.160 |
| *ETFA* | 10 | ? | 0.714 | 0.032 |
| *MTMR14* | 12 | 12019 | 0.771 | 0.387 |
| *EGR1* | 13 | 20 | 0.696 | 0.090 |
| *PITPNC1* | 18 | 3145 | 0.512 | 0.065 |
| *PECAM1* | 18 | 3437 | 0.804 | 0.468 |
| *CLIP2* | 19 | 3613 | 0.588 | 0.048 |
| *NCOA3* | 20 | 15612 | 0.759 | 0.043 |
| ^a^*HypPC2*: Hypothetical protein LOC100221932 (zebra finch) (chromosome 2)  ^b^*HypPC6*: hypothetical protein LOC100223253 (zebra finch) (chromosome 6) | | | | |
