# Supplementary material for: Evidence for Mito-Nuclear and Sex-Linked Reproductive Barriers between the Hybrid Italian Sparrow and Its Parent Species
Source: PLoS Genet. 2014 Jan 9;10(1):e1004075. doi: 10.1371/journal.pgen.1004075 (PMC3886922; doi:10.1371/journal.pgen.1004075)
Supplement: Supplementary file 5 [file pgen.1004075.s002.doc]

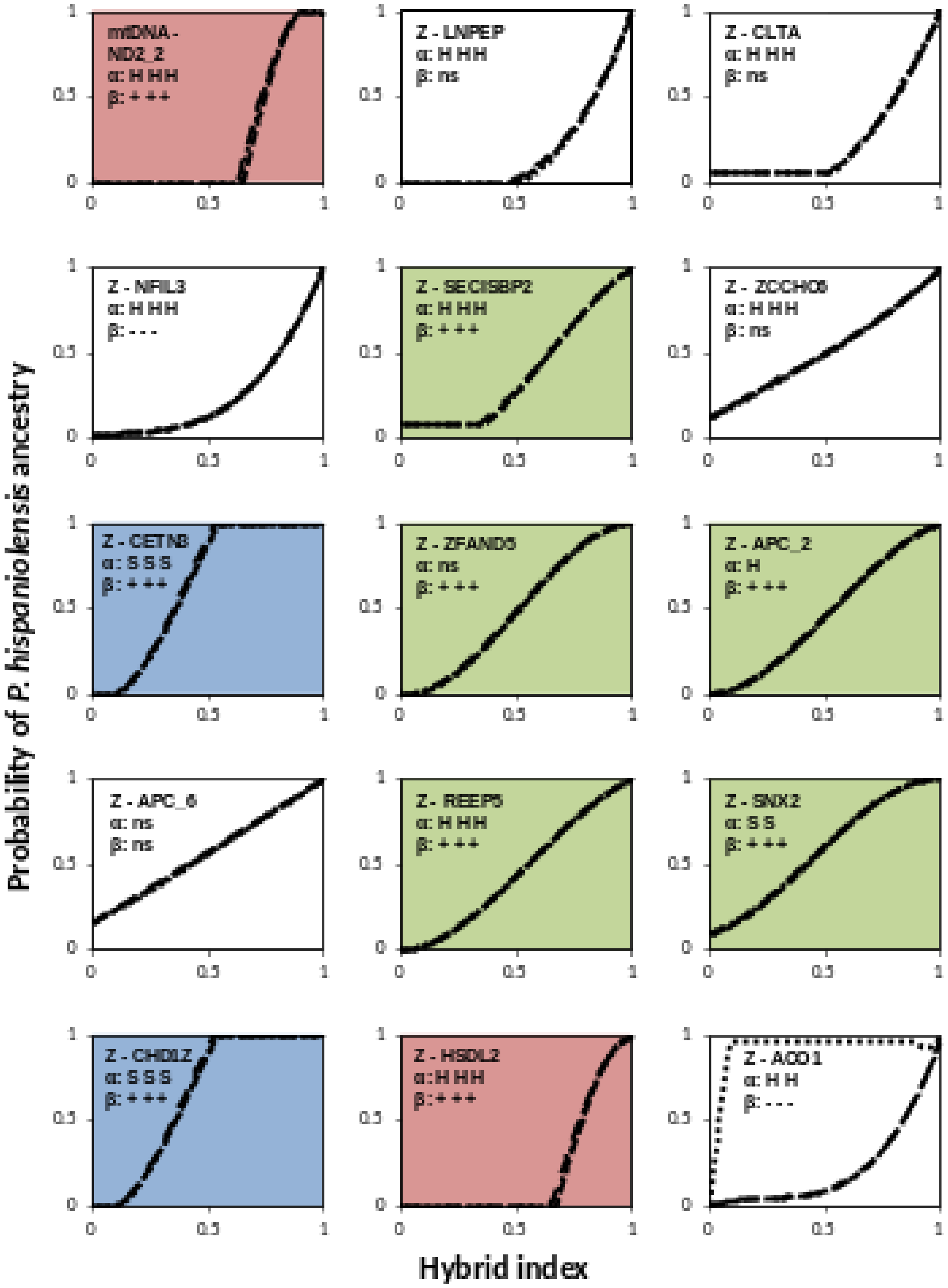
mtDNA - ND2_2
α: H H H
β: + + +
Z - LNPEP
α: H H H
β: ns
Z - CLTA
α: H H H
β: ns
Z - NFIL3
α: H H H
β: - - -
Z - SECISBP2
α: H H H
β: + + +
Z - ZCCHC6
α: H H H
β: ns
Z - CETN3
α: S S S
β: + + +
Z - ZFAND5
α: ns
β: + + +
Z - APC_2
α: H
β: + + +
Z - APC_6
α: ns
β: ns
Z - REEP5
α: H H H
β: + + +
Z - SNX2
α: S S
β: + + +
Z - CHD1Z
α: S S S
β: + + +
Z - HSDL2
α: H H H
β: + + +
Z - ACO1
α: H H
β: - - -
Probability of P. hispaniolensis ancestry
Hybrid index

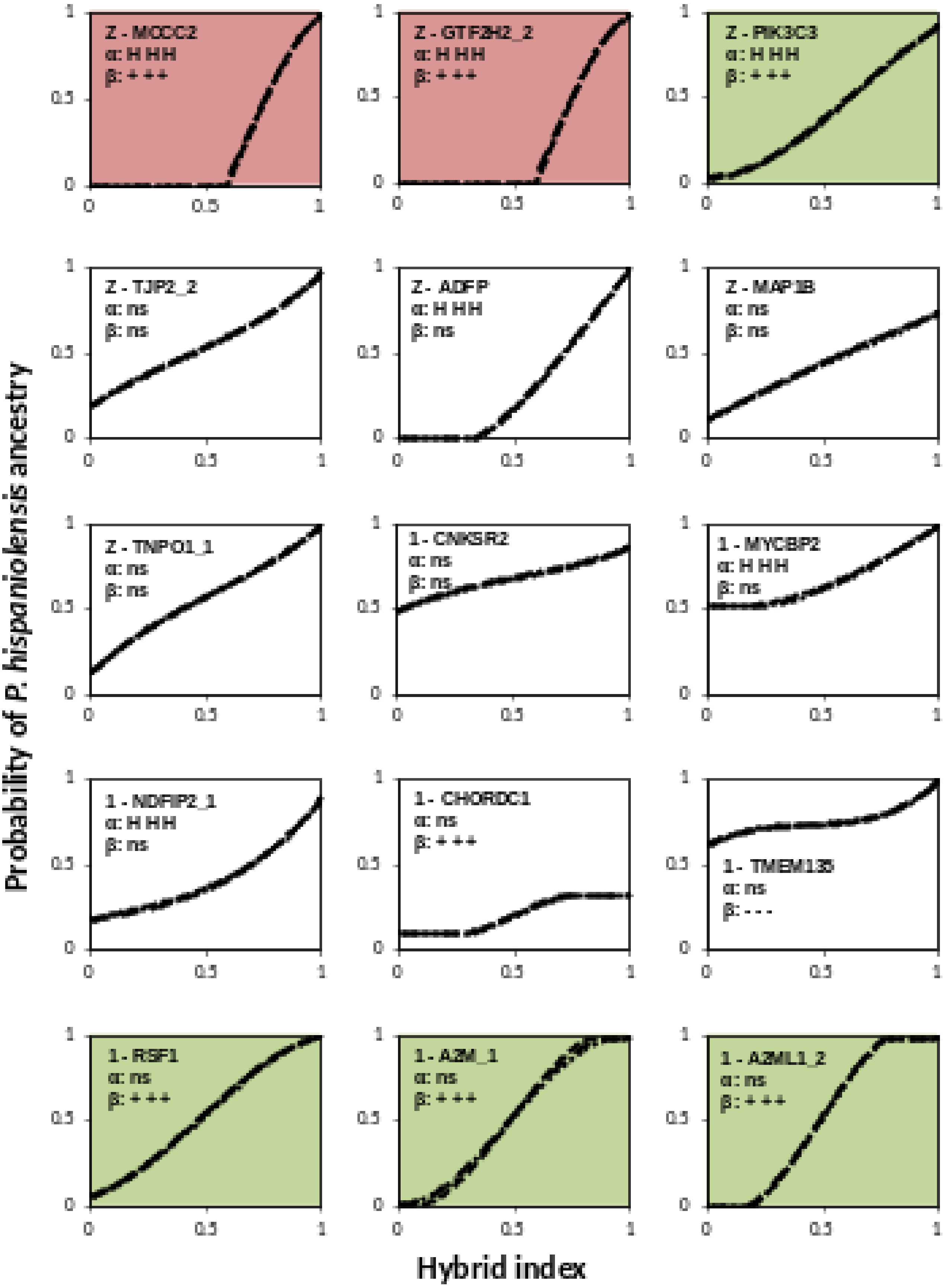
Z - MCCC2
α: H H H
β: + + +
Z - GTF2H2_2
α: H H H
β: + + +
Z - PIK3C3
α: H H H
β: + + +
Z - TJP2_2
α: ns
β: ns
Z - ADFP
α: H H H
β: ns
Z - MAP1B
α: ns
β: ns
Z - TNPO1_1
α: ns
β: ns
1 - CNKSR2
α: ns
β: ns
1 - MYCBP2
α: H H H
β: ns
1 - NDFIP2_1
α: H H H
β: ns
1 - CHORDC1
α: ns
β: + + +
1 - TMEM135
α: ns
β: - - -
1 - RSF1
α: ns
β: + + +
1 - A2M_1
α: ns
β: + + +
1 - A2ML1_2
α: ns
β: + + +
Probability of P. hispaniolensis ancestry
Hybrid index

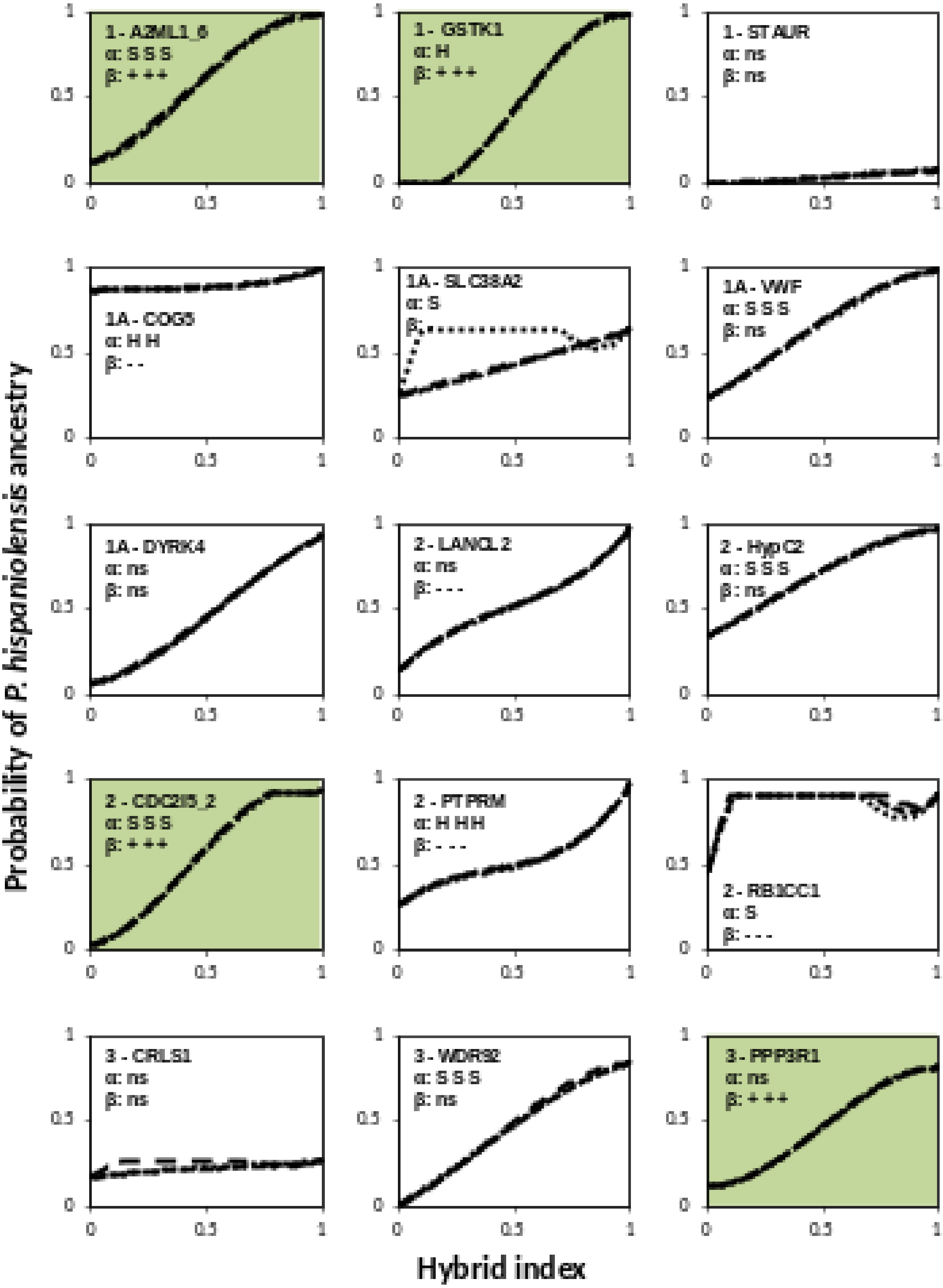

Probability of P. hispaniolensis ancestry
Hybrid index
1 - A2ML1_6
α: S S S
β: + + +
1 - GSTK1
α: H
β: + + +
1 - STAUR
α: ns
β: ns
1A - COG5
α: H H
β: - -
1A - SLC38A2
α: S
β:
1A - VWF
α: S S S
β: ns
1A - DYRK4
α: ns
β: ns
2 - LANCL2
α: ns
β: - - -
2 - HypC2
α: S S S
β: ns
2 - CDC2l5_2
α: S S S
β: + + +
2 - PTPRM
α: H H H
β: - - -
2 - RB1CC1
α: S
β: - - -
3 - CRLS1
α: ns
β: ns
3 - WDR92
α: S S S
β: ns
3 - PPP3R1
α: ns
β: + + +

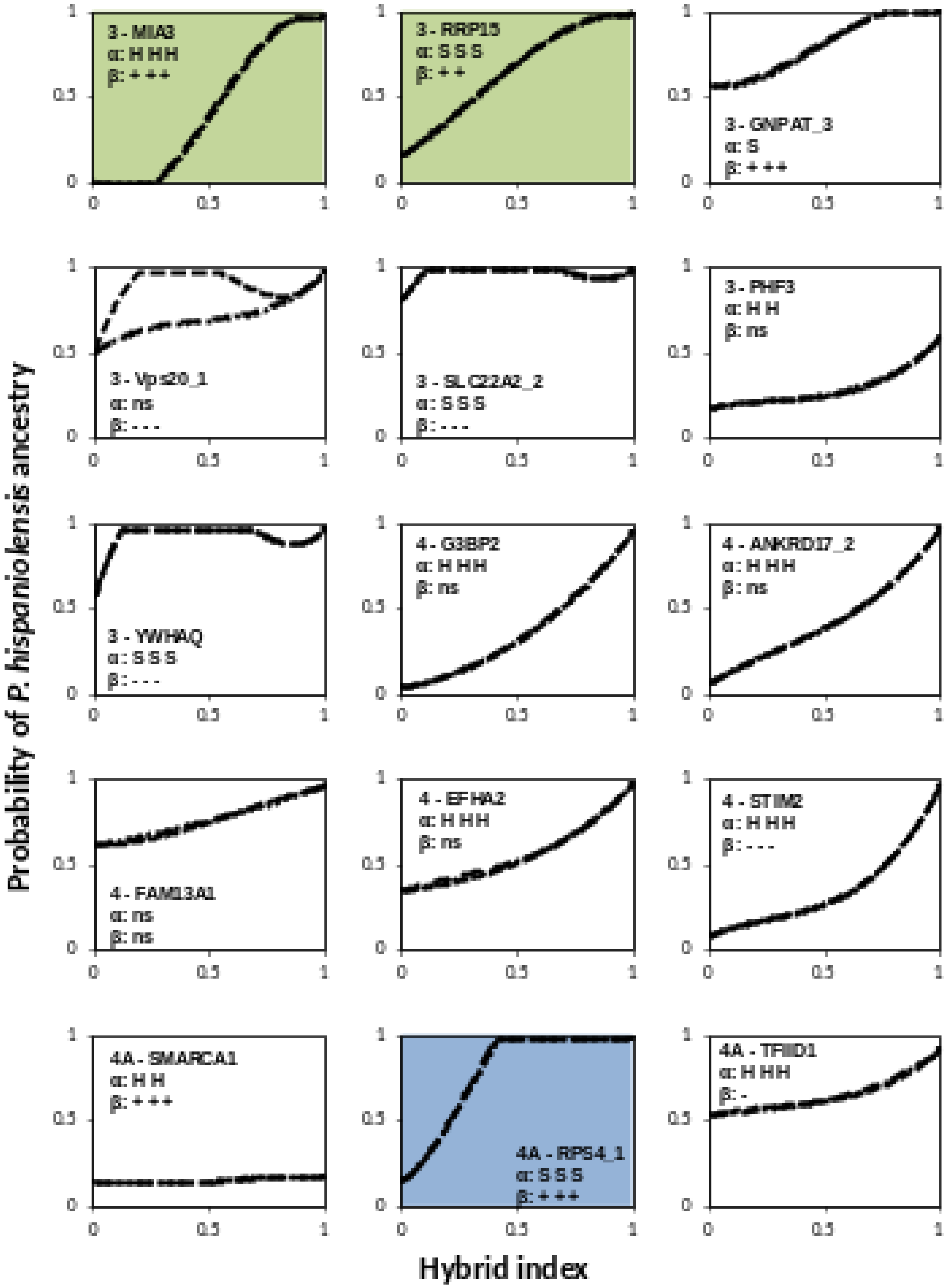
Probability of P. hispaniolensis ancestry
Hybrid index
3 - MIA3
α: H H H
β: + + +
3 - RRP15
α: S S S
β: + +
3 - GNPAT_3
α: S
β: + + +
3 - Vps20_1
α: ns
β: - - -
3 - SLC22A2_2
α: S S S
β: - - -
3 - PHF3
α: H H
β: ns
3 - YWHAQ
α: S S S
β: - - -
4 - G3BP2
α: H H H
β: ns
4 - ANKRD17_2
α: H H H
β: ns
4 - FAM13A1
α: ns
β: ns
4 - EFHA2
α: H H H
β: ns
4 - STIM2
α: H H H
β: - - -
4A - SMARCA1
α: H H
β: + + +
4A - RPS4_1
α: S S S
β: + + +
4A - TFIID1
α: H H H
β: -

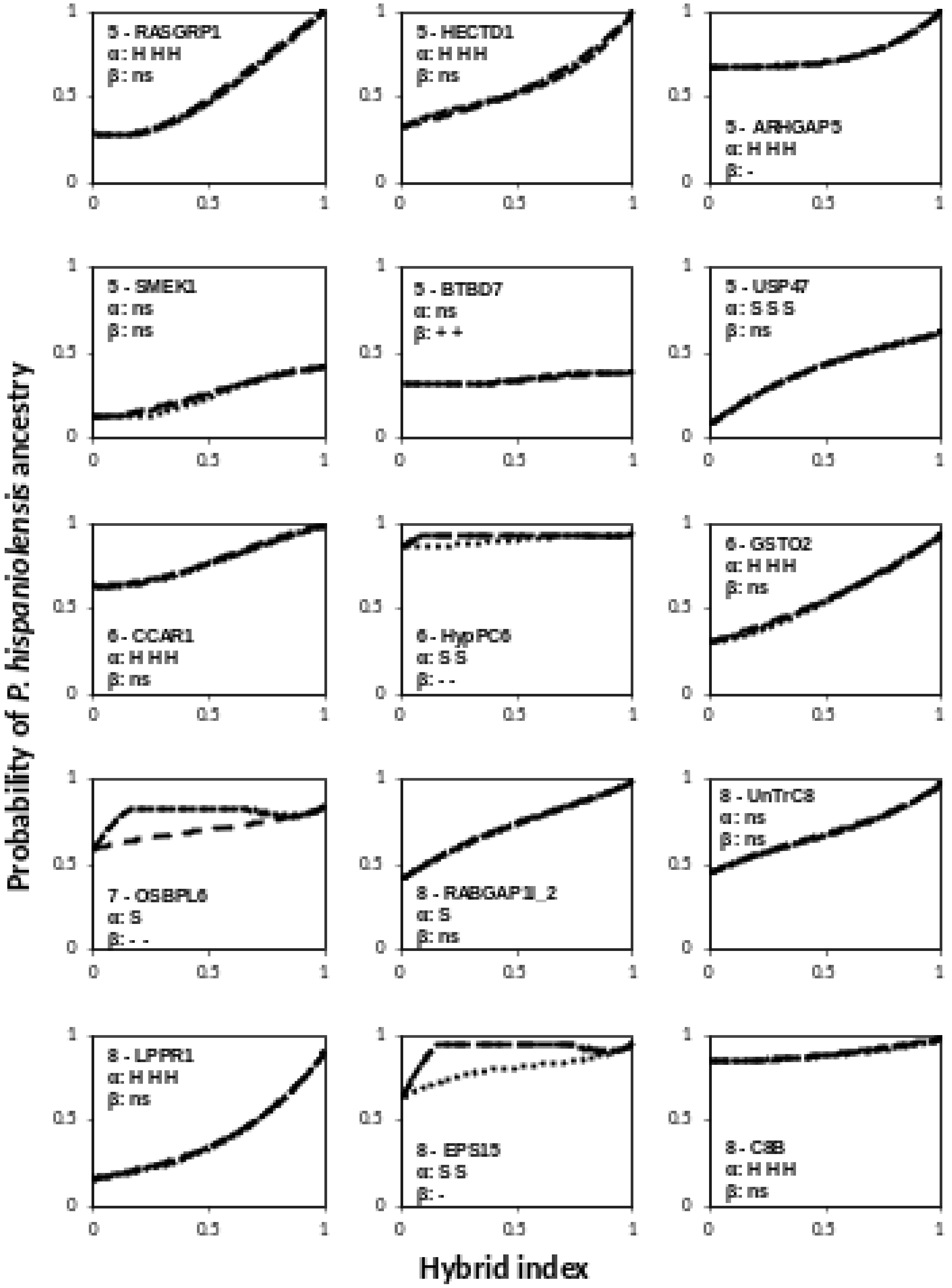

Probability of P. hispaniolensis ancestry
5 - RASGRP1
α: H H H
β: ns
5 - HECTD1
α: H H H
β: ns
5 - ARHGAP5
α: H H H
β: -
5 - SMEK1
α: ns
β: ns
5 - BTBD7
α: ns
β: + +
5 - USP47
α: S S S
β: ns
6 - CCAR1
α: H H H
β: ns
6 - HypPC6
α: S S
β: - -
6 - GSTO2
α: H H H
β: ns
7 - OSBPL6
α: S
β: - -
8 - RABGAP1L_2
α: S
β: ns
8 - UnTrC8
α: ns
β: ns
8 - LPPR1
α: H H H
β: ns
8 - EPS15
α: S S
β: -
8 - CSB
α: H H H
β: ns
Hybrid index

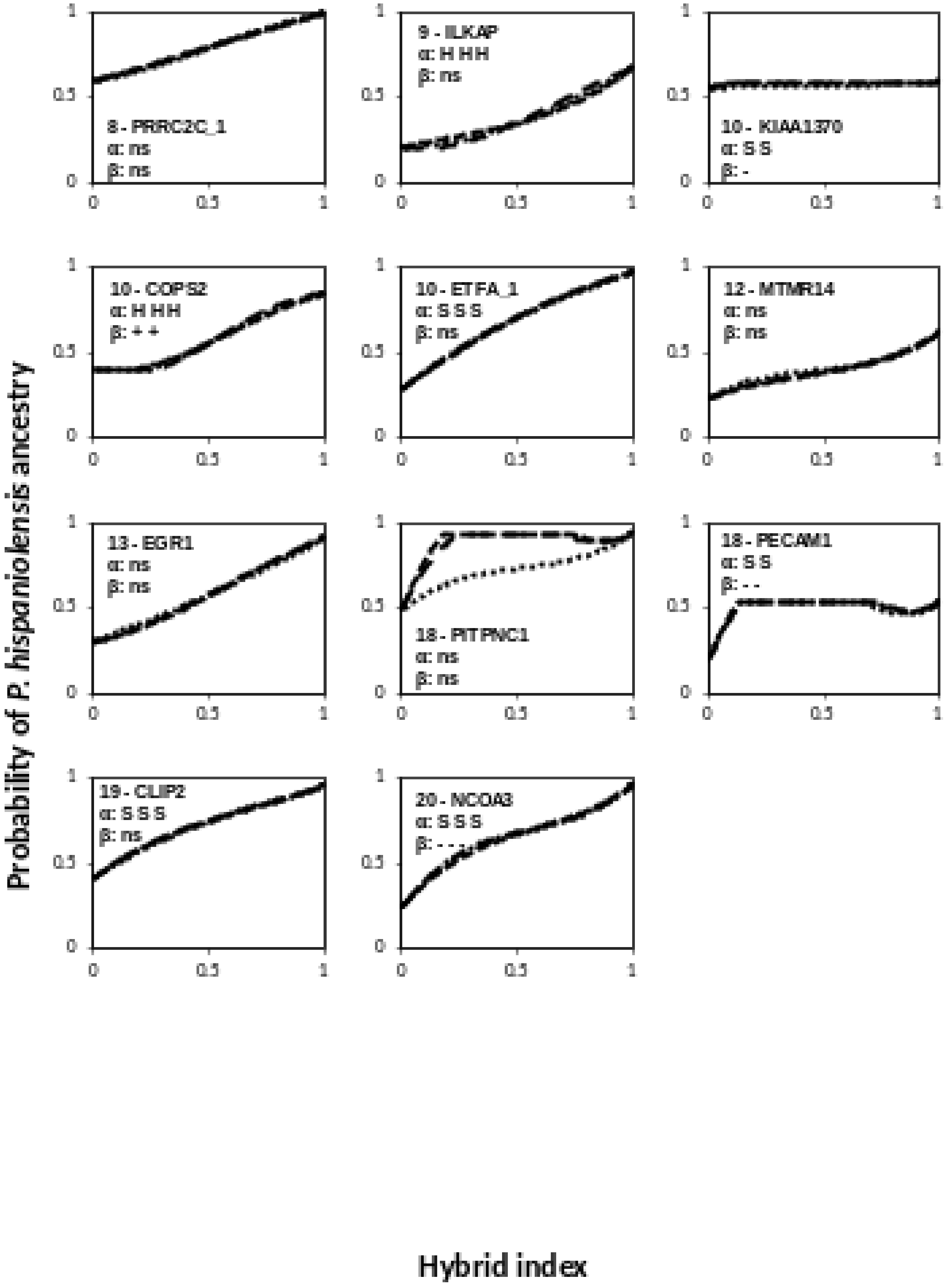
8 - PRRC2C_1
α: ns
β: ns
9 - ILKAP
α: H H H
β: ns
10 - KIAA1370
α: S S
β: -
10 - COPS2
α: H H H
β: + +
10 - ETFA_1
α: S S S
β: ns
12 - MTMR14
α: ns
β: ns
13 - EGR1
α: ns
β: ns
18 - PITPNC1
α: ns
β: ns
18 - PECAM1
α: S S
β: - -
19 - CLIP2
α: S S S
β: ns
20 - NCOA3
α: S S S
β: - -
Probability of P. hispaniolensis ancestry
Hybrid index
