# Supplementary material for: Evidence for Mito-Nuclear and Sex-Linked Reproductive Barriers between the Hybrid Italian Sparrow and Its Parent Species
Source: PLoS Genet. 2014 Jan 9;10(1):e1004075. doi: 10.1371/journal.pgen.1004075 (PMC3886922; doi:10.1371/journal.pgen.1004075)
Supplement: Supplementary file 6 [file pgen.1004075.s004.doc]

Locus	Marker position	LNPEP	CLTA	NFIL3	SEC1SBP2	CETN3	ZFAND5	APC_2	APC_6	REEP5	SNX2	CHD1Z	HSDL2	ACO1	MCCC2	GTF2H2	PIK3C3
**LNPEP**	1650																
**CLTA**	2048	398															
**NFIL3**	6415	4765	4367														
**SEC1SBP2**	7215	5565	5167	800													
**CETN3**	12839	11189	10791	6424	5624												
**ZFAND5**	16367	14717	14319	9952	9152	3528											
**APC_2**	21208	19558	19160	14793	13993	8369	4841										
**APC_6**	21208	19558	19160	14793	13993	8369	4841	0									
**REEP5**	21269	19619	19221	14854	14054	8430	4902	61	61								
**SNX2**	23313	21663	21265	16898	16098	10474	6946	2105	2105	2044							
**CHD1Z**	24761	23111	22713	18346	17546	11922	8394	3553	3553	3492	1448						
**HSDL2**	25608	23958	23560	19193	18393	12769	9241	4400	4400	4339	2295	847					
**ACO1**	31875	30225	29827	25460	24660	19036	15508	10667	10667	10606	8562	7114	6267				
**MCCC2**	32227	30577	30179	25812	25012	19388	15860	11019	11019	10958	8914	7466	6619	352			
**GTF2H2**	32332	30682	30284	25917	25117	19493	15965	11124	11124	11063	9019	7571	6724	457	105		
**PIK3C3**	34838	33188	32790	28423	27623	21999	18471	13630	13630	13569	11525	10077	9230	2963	2611	2506	
**ADFP**	58329	56679	56281	51914	51114	45490	41962	37121	37121	37060	35016	33568	32721	26454	26102	25997	23491
