# Supplementary material for: Evidence for Mito-Nuclear and Sex-Linked Reproductive Barriers between the Hybrid Italian Sparrow and Its Parent Species
Source: PLoS Genet. 2014 Jan 9;10(1):e1004075. doi: 10.1371/journal.pgen.1004075 (PMC3886922; doi:10.1371/journal.pgen.1004075)
Supplement: Supplementary file 7 [file pgen.1004075.s005.doc]

A

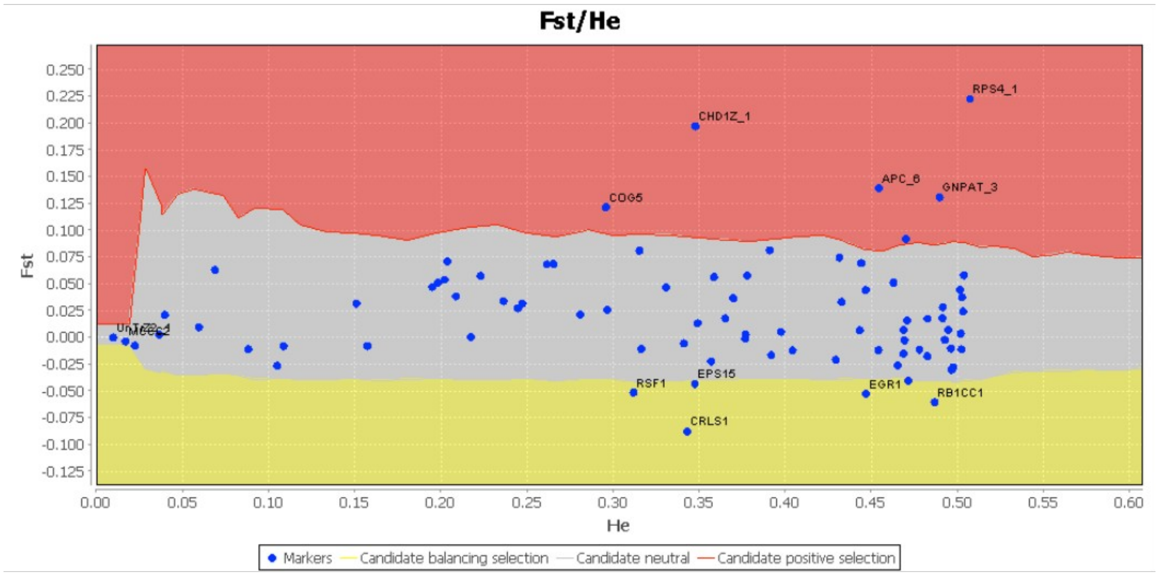
Fst/He
Fst
He
RPS4_1
CHD1Z_1
APC_6
GNPAT_3
COG5
RSF1
EPS15
EGR1
RB1CC1
CRLS1
Markers
Candidate balancing selection
Candidate neutral
Candidate positive selection

B

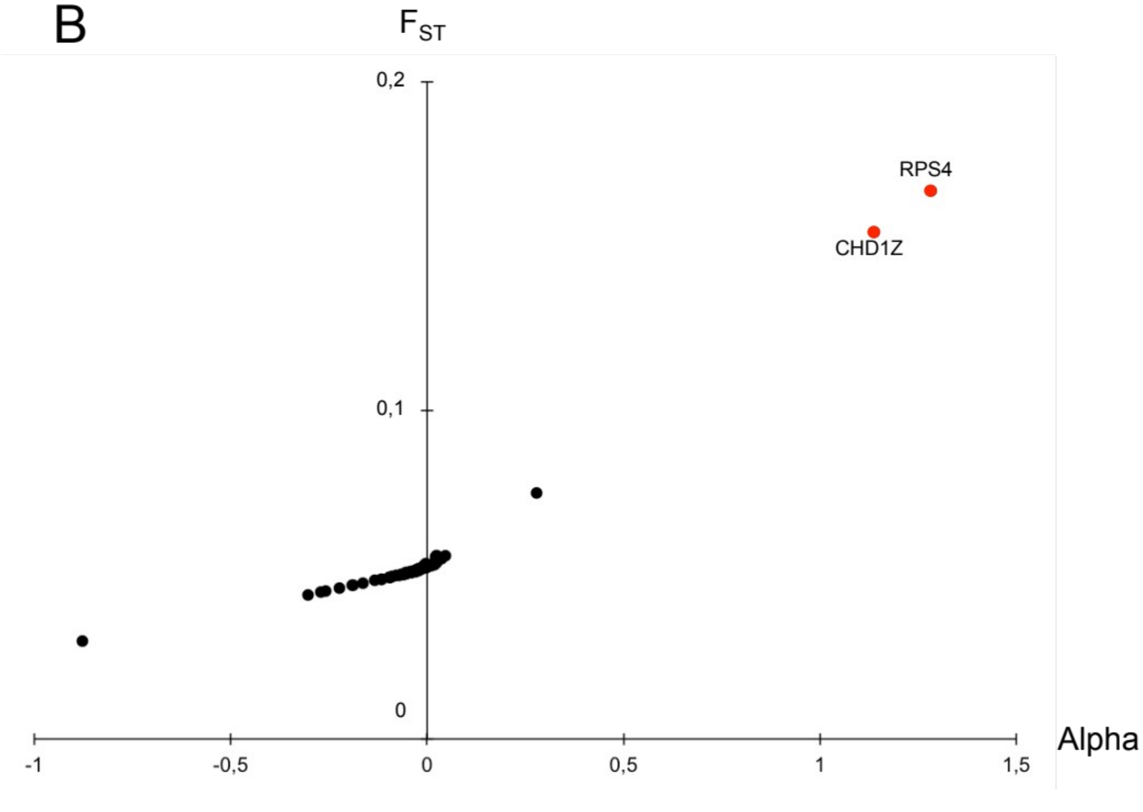
F_{ST}
RPS4
CHD1Z
Alpha
